# Nicotinamide mononucleotide stimulates the activity of bursting slow-oscillation neurons in the supramammillary nucleus and enhances REM sleep

**DOI:** 10.1016/j.isci.2026.115741

**Published:** 2026-04-15

**Authors:** Youssouf Cissé, Cynthia S. Brace, Virginia Hsu, Carla M. Yuede, Nicholas Rensing, Michael Wong, Shin-ichiro Imai

**Affiliations:** 1 Department of Developmental Biology, Washington University School of Medicine, Saint Louis, Missouri, USA; 2 Department of Psychiatry, Washington University School of Medicine, Saint Louis, Missouri, USA; 3 Department of Neurology, Washington University School of Medicine, Saint Louis, Missouri, USA; 4 Department of Medicine, Washington University School of Medicine, Saint Louis, Missouri, USA

**Keywords:** neuroscience

## Abstract

Nicotinamide mononucleotide (NMN) plays an important role in enhancing NAD^+^ levels and maintaining tissue functions in both mice and humans. Here, we show that NMN stimulates GABAergic neurons expressing *Slc12a8*, an NMN transporter, in the supramammillary (SuM) nucleus and enhances rapid eye movement (REM) sleep. By juxtacellular recording in the SuM, we have identified bursting slow-oscillation (SO) neurons that discharge in rhythmic theta burst at maximal rates during REM. Their firing frequency significantly decreases in aged mice, and NMN restores it to the level of young mice. The *Slc12a8*-deficient mice show defects in NMN response, decreased percent theta power during REM sleep, and impaired novel object recognition. Optogenetic stimulation of the SuM GABAergic neurons projecting to the cortex triggers cortical SO and theta rhythm, whereas chemogenetic inhibition of those neurons predominantly decreases REM sleep. These findings suggest that NMN stimulates SuM bursting SO neurons and promotes REM sleep during aging.

## Highlights

• The supramammillary nucleus (SuM) contains bursting slow-oscillation (SO) neurons
• Nicotinamide mononucleotide (NMN) stimulates bursting SO neurons and REM sleep
• *Slc12a8*-KO mice exhibit impairments in NMN response and novel object recognition
• SuM *Slc12a8*-positive, GABAergic neurons trigger SO and theta activity in the cortex

## Graphical abstract

**Figure:**
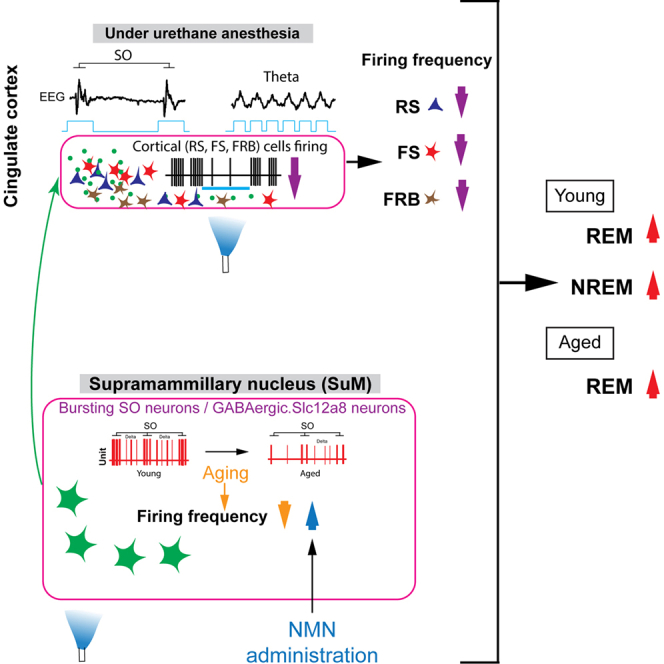

## Introduction

With global populational aging, more older adults suffer from sleep-related problems. In the US, approximately one in three adults reportedly does not have enough sleep every day, and 50–70 million people are estimated to have chronic or ongoing sleep problems.^1^ In adults, total sleep time, sleep efficiency, percentages of slow-wave and rapid eye movement (REM) sleep, and REM latency significantly decrease with age.^2^ In particular, decreased percentage REM sleep is associated with greater risks of all-cause mortality and cardiovascular and other non-cancer-related mortality rates.^3^ What causes such age-associated changes in sleep still remains poorly understood. Most recently, PR-domain containing protein 13 (*Prdm13*)-positive neurons in the dorsomedial hypothalamus (DMH) have been identified and demonstrated to cause age-associated sleep alterations, such as sleep fragmentation and increased sleep attempts in mice.^4^ Interestingly, overexpression of *Prdm13* in the aged DMH partially rescues age-associated sleep problems.^4^ Additionally, *Prdm13* expression is upregulated in the aged DMH by increasing circulating levels of extracellular nicotinamide phosphoribosyltransferase (eNAMPT), a key NAD^+^ biosynthetic enzyme in mammals, implicating a connection between age-associated changes in NAD^+^ metabolism and sleep.^5^

Recently, it has become a consensus that decreased NAD^+^ availability at a systemic level is a critical driving force that causes age-associated functional decline.^6^^,^^7^^,^^8^^,^^9^ During aging, multiple tissues, including the hypothalamus, show significant decreases in NAD^+^ levels,^7^^,^^10^ and thus, a concept of “NAD^+^ boosting” has been extensively tested as a potential anti-aging intervention, mainly using NAD^+^ intermediates, such as NMN and nicotinamide riboside (NR).^6^^,^^11^ In rodents, many studies have already demonstrated remarkable effects of these NAD^+^ intermediates to mitigate age-associated functional decline and ameliorate symptoms of age-associated diseases.^7^^,^^8^^,^^9^^,^^11^ In humans, some promising efficacies have been reported in clinical trials: NMN improves skeletal muscle insulin sensitivity,^6^ aerobic capacity during exercise,^12^ and gate speed and left grip strength,^13^ whereas NR increases percentage fat-free mass and sleeping metabolic rate,^14^ alters cerebral metabolism in Parkinson’s disease patients,^15^ and reduces inflammation.^15^^,^^16^

When assessing the effect of NMN on brain activity, we initially made an unexpected finding that NMN was able to induce recurrent rhythmic activity at theta (3–6 Hz) frequency in anesthetized mice. Thus, we aimed to study (1) whether NMN can enhance REM sleep by activating a certain specific neuronal subset and (2) how NMN and its transporter Slc12a8 contribute to the generation of REM sleep during aging. Indeed, we found that NMN enhanced REM sleep in both unanesthetized young and aged mice. These findings led us to a discovery of a previously unidentified neuronal population in the supramammillary (SuM) region. By using the juxtacellular recording of single neuronal activity, we identified bursting neurons that discharged in slow oscillation (SO) with highest activity during REM sleep. Whereas their firing frequency significantly decreased in aged mice, NMN was able to restore the firing frequency to the level of young mice. Consistently, the mice deficient in *Slc12a8*, an NMN transporter, showed defects in NMN response, decreased percent theta power during REM sleep, and impaired novel object recognition. Interestingly, whereas optogenetic stimulation of the SuM GABAergic neurons, which project to the cingulate cortex (CC), triggered cortical SO and theta rhythm, recapitulating the effect of NMN, chemogenetic inhibition of the SuM GABAergic neurons significantly decreased REM sleep in young mice. These findings demonstrate a critical role of NMN in stimulating SuM bursting SO neurons and promoting REM sleep during aging.

## Results

### NMN induces transient and recurrent EEG activations in anesthetized mice

**Figure 1:**
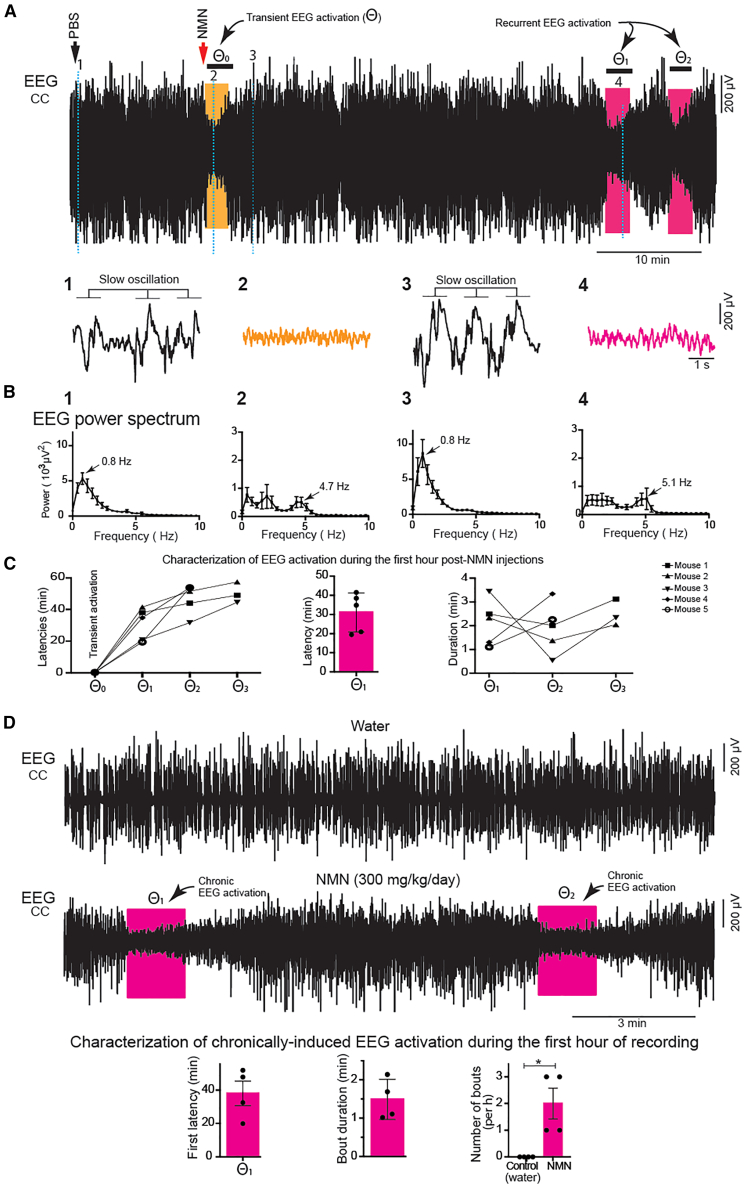
NMN induces recurrent EEG activation in anesthetized mice (A) EEG recording during PBS administration (i.p., black arrow) followed by NMN administration (i.p., red arrow). Segments 1, 2, 3, and 4 marked on the EEG traces are expanded below to show slow oscillation (segment 1), the transient activation (segment 2, orange color, θ_0_), recovery to slow oscillation (segment 3), and the first spontaneous recurrent activation (segment 4, pink color, θ_1_). (B) The corresponding mean EEG power spectrum of each segment is plotted (*n* = 5), and the peak frequencies are indicated by arrows. (C) Latencies of EEG activation events during an hour post NMN administration (left), latency to first recurrent EEG activation across mice (middle), and durations of recurrent activation events (right). (D) Chronic NMN administration (see STAR Methods). The EEGs of control (water given, top) and NMN-administered (300 mg/kg/day, 4 weeks, bottom) mice are shown. Two EEG activation (θ_1_ and θ_2_) events (arrows) are indicated in NMN-administered mice. The characterization of EEG activation including latency (left), bout duration (middle) and number of bouts (right, bottom, *n* = 4, Student’s unpaired *t* test, ∗*p* < 0.05). Results are presented as mean ± SEM.

**Figure 2:**
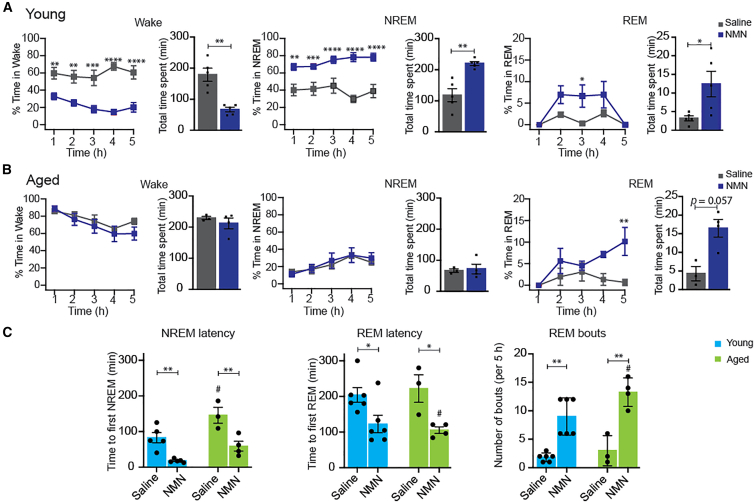
NMN enhances NREM/REM sleep in young mice and REM sleep in aged mice (A and B) The percentages of wake, NREM, and REM sleep and the total time spent in each state in unanesthetized young (A) and aged (B) mice are shown. (∗*p* < 0.05, ∗∗*p* < 0.01, ∗∗∗*p* < 0.001, ∗∗∗∗*p* < 0.0001 for young [saline] vs. young [NMN] and aged [saline] vs. aged [NMN]; two-way repeated measures ANOVA with Tukey’s post hoc test and Student’s *t* test, *n* = 5–6 young mice; *n* = 3–4 aged mice per group). (C) The latency of NREM (left) and REM (middle) sleep, and REM bouts (right) of young and aged mice are shown. (∗*p* < 0.05, ∗∗*p* < 0.01 for young [saline] vs. young [NMN] and aged [saline] vs. aged [NMN]; ^#^*p* = 0.05 for young [saline] vs. aged [saline] and young [NMN] vs. aged [NMN]; one-way ANOVA with Tukey’s post hoc test, *n* = 5–6 young mice; *n* = 3–4 aged mice per group). Results are presented as mean ± SEM.

**Figure 3:**
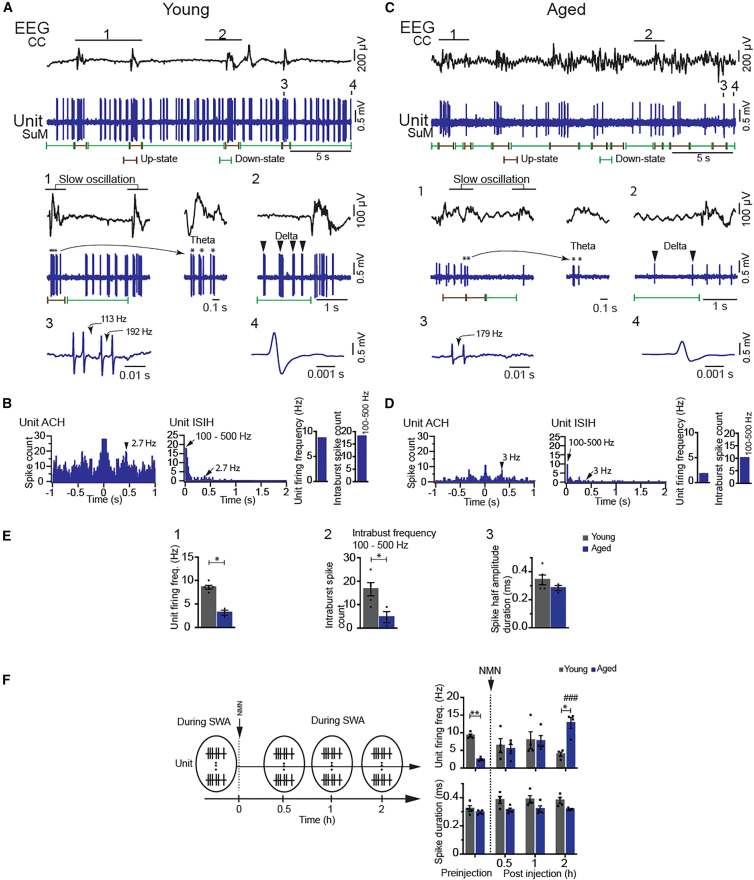
NMN restores the unit firing frequency of bursting slow-oscillation neurons in the SuM of anesthetized aged mice (A–D) EEG and the unit activity are displayed in young (A and B) and aged (C and D) mice. Segments 1, 2, 3, and 4 marked on the EEG traces are expanded below. In segment 1 (left), the unit displays the phasic discharge of bursts (asterisks). The trace of segment 1 is further expanded (right) to capture the bursting activity at theta (∼7 Hz) frequency. Segment 2 displays a part of the inactive period where the unit discharges at delta (∼3 Hz) frequency as indicated by arrowheads. The expanded traces of segments 3 and 4 show a single burst event and a single spike, respectively. The autocorrelation histogram (ACH) (left), interspike interval histogram (ISIH) (middle) of the units (A and C), and their firing rates and intraburst spike counts of 100–500 Hz and 100 Hz frequencies (right) are shown for young (B) and aged (D) mice. (E) Unit spike firing (1), intraburst spike count (2), and spike half amplitude duration (3) are compared between young and aged mice. (∗*p* < 0.05, unpaired *t* test, *n* = 5 from 5 young mice; *n* = 3 from 3 aged mice). (F) (Left) The scheme of the recordings before and after NMN administration. (Right) Unit firing frequency (top) and spike duration (bottom) before and after NMN administration are compared between young and aged mice (∗*p* < 0.05, ∗∗*p* < 0.01, ^###^*p* < 0.001 [0 time point vs. 2 h time point in aged mice], two-way repeated measures ANOVA with Šidák post hoc test, *n* = 4 from 4 young and *n* = 11 from 4 aged mice for pre-injection, and *n* = 24 from 4 young and *n* = 25 from 4 aged mice for NMN-administered neurons; 1–5 neurons [units] were recorded per mouse. When multiple cells were recorded, values were averaged per mouse [scheme, left] see STAR Methods). Results are presented as mean ± SEM.

**Figure 4:**
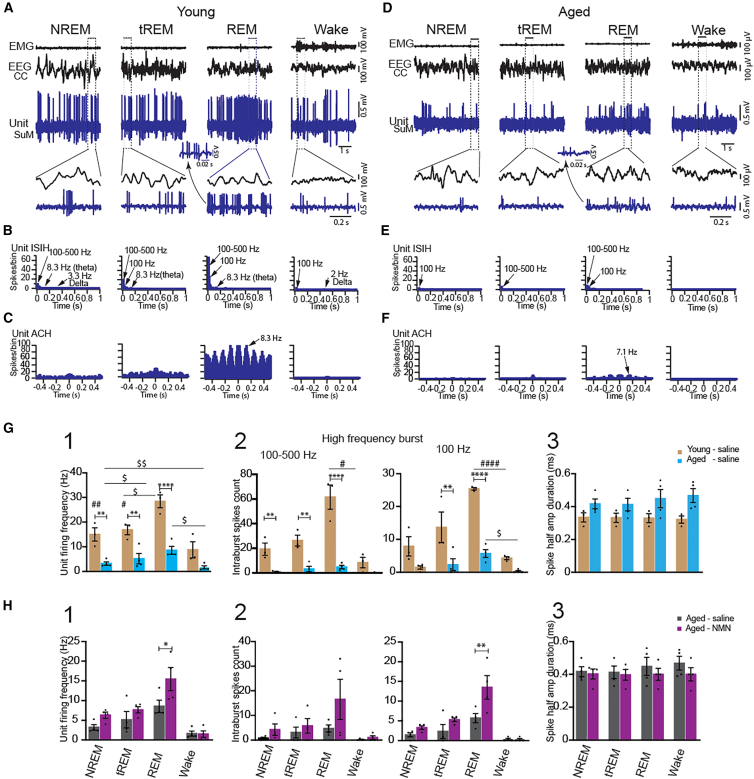
The activity of the bursting NREM/REM-active SO neurons in the SuM is significantly rescued by NMN in aged mice (A–F) Discharge profiles of bursting slow oscillation neurons in association with EMG and EEG recordings for 5s epochs during NREM, tREM, REM and wake in young (A–C) and aged (D–F) mice. The unit ISIH and ACH are displayed for young (B and C) and aged (E and F) mice, respectively. Note that the unit ACH displays rhythmicity in it during REM in young (C) and aged (F) mice. (G and H) The unit firing frequency (1), intraburst spike counts at 100–500 Hz and 100 Hz (2), and spike half amplitude duration (3) are compared after saline administration between young and aged mice (G) or between saline and NMN administration in aged mice (H). (young saline, *n* = 3 ^#^*p* < 0.05, ^##^*p* < 0.01, ^####^*p* < 0.0001 from 3 mice; aged saline, *n* = 4 from 4 mice, ^$^*p* < 0.05, ^$$^*p* < 0.01; comparing NREM vs. REM, tREM vs. REM, and wake vs. REM, one-way ANOVA with Tukey’s post hoc test; young vs. aged saline and aged saline vs. aged-NMN, ∗*p* < 0.05, ∗∗*p* < 0.01, ∗∗∗∗*p* < 0.0001, two-way ANOVA with Šidák post hoc test, *n* = 4 for Aged-NMN from 4 mice). Results are presented as mean ± SEM.

In order to examine the global effect of NMN on brain activity, we recorded electroencephalogram (EEG) under deep urethane anesthesia before and after NMN administration. During continuous EEG recording in urethane-anesthetized young mice, PBS was first administered intraperitoneally (i.p.), followed by NMN i.p. administration ∼10 min later. Compared to PBS (Figure 1A, segment 1; Figure 1B, 1), NMN administration induces an immediate, transient EEG activation (θ_0_, Figure 1A, segment 2; Figure 1B, 2), followed by a gradual EEG recovery to slow-wave activity (SWA) (Figure 1A, segment 3; Figure 1B, 3). NMN administration was associated with significant increase of slow (0.2–1 Hz) oscillation (SO) at 0–5 min time points and theta (3–6 Hz) peak power at 5–10 min time points (Figures S1A and S1C). Delta (1–3 Hz) peak power also showed a trend of an increase (Figure S1B). This EEG activation observed immediately after NMN administration may not reflect a direct central effect of NMN because it is too fast, and it may be due to some peripheral sensory stimulation caused by NMN. Interestingly, after a delay of ∼34 min following NMN administration, EEG shift from irregular SWA to rhythmic cortical EEG activation was observed, peaking at 5.1 Hz of theta frequency, thus hereafter termed recurrent EEG activation (Figure 1A, segment 4; Figure 1B, 4). The mean latency (θ_1_; Figure 1C, left and middle graphs), the mean duration (θ_1_–θ_3_; Figure 1C, right graph), and the bout frequency of the recurrent EEG activation were 31.0 ± 4.6 min, 2.1 ± 0.2 min, and 2.6 ± 0.2 times per hour, respectively. We also examined the effect of chronic administration of NMN on EEG activation during the first hour of recording in urethane-anesthetized young mice after a 4-week-long NMN treatment (300 mg/kg/day, see STAR Methods). We observed EEG activation in the NMN-treated group, resembling the pattern observed under acute NMN administration, whereas no EEG activation was detected in control (water given) mice (Figure 1D, top and middle). The latency, duration, and bout frequency of recurrent EEG activation were measured and plotted (Figure 1D, bottom). The NMN-treated group showed a significantly higher number of EEG activation bouts compared to controls (Figure 1D, bottom right). These findings provide initial evidence implicating that NMN may stimulate recurrent theta-frequency cortical activation and could potentially modulate REM-related activity even under the natural sleep-wake cycle.

### NMN enhances REM sleep in both unanesthetized young and aged mice

To determine whether NMN administration indeed enhances REM sleep, we next recorded EEG and EMG activities in unanesthetized head-fixed young and aged mice. Saline and NMN were administered at least ∼10 min prior to EEG recording (see STAR Methods). Whereas saline-administered young mice sustained waking (>60%) during a 5-h recording time (Figure 2A, left), NMN-administered young mice showed significant decreases in wake (20%–40%) and significant increases in non-REM (NREM) sleep (60%–80%) during a 5-h recording time (Figure 2A, middle). More importantly, NMN-administered young mice displayed increases in REM sleep through 2–4 h time points, with statistical significance at 3 h time point, and a significant increase in the total time spent in REM sleep (Figure 2A, right). Indeed, during wake, NMN-treated young mice exhibited significantly shorter episode durations and higher numbers of bouts, compared to the saline-treated control mice (Figure S2A, left two images), implying that NMN treatment decreases wakefulness. NMN-treated young mice also showed increased NREM episode durations, with statistical significance at 3h time point, but no significant differences in its bouts (Figure S2A, middle two images). Remarkably, NMN-treated young mice displayed a gradual increase in REM episode durations from 2 to 5-h post-injection to reach significance at 3-h time point, compared to saline-treated controls, and the numbers of REM bouts were also higher in the NMN group at 3–5 h, with statistical significance at 3-h time point (Figure S2A, right two images). These results suggest that NMN administration in young mice promotes consolidated sleep architecture, characterized by reduced wakefulness and increased stability of NREM and REM sleep.

Contrary to young mice, NMN administration did not alter wake and NREM sleep during a 5-h recording time in aged mice (Figure 2B, left and middle). However, NMN increased percentage of REM sleep at the 4–5-h time points, particularly showing a statistically significant difference at the 5-h time point. Additionally, the total time spent in REM sleep tended to be increased in aged mice (Figure 2B, right). Whereas episode durations and numbers of bouts for wake and NREM were comparable between NMN-treated and saline-treated aged mice at all time points (Figure S2B, left and middle images), episode durations and numbers of bouts for REM exhibited a trend of increase from 2 to 5-h time points in NMN-treated aged mice, compared to saline controls (Figure S2B, right two panels), suggesting that the REM-increasing effect of NMN is more prominent, compared to those on wake and NREM, in aged mice.

Between young and aged mice, the latency to NREM sleep (continuous 30 s deep sleep) was significantly reduced in both NMN-administered young and aged mice (Figure 2C, left), whereas it increased in aged mice, compared to young mice, in both saline and NMN administrations. REM latency also showed significant decreases in both young and aged mice (Figure 2C, middle). Furthermore, REM bouts were significantly increased in both NMN-administered young and aged mice, compared to saline-administered mice (Figure 2C, right). There were no significant differences in spectral power across wake, NREM, and REM states in both young and aged mice (Figures S2C and S2D).

### Bursting SO neurons in the SuM respond to NMN in anesthetized aged mice

We next examined which area of the brain was stimulated by NMN. We immunostained c-Fos on brain sections of young mice 90 min after NMN administration. Interestingly, we found significant increases in c-Fos signals in the anterior part of the SuM, particularly around bregma −2.45 mm, in NMN-administered mice (Figure S3A). We also observed c-Fos signals in the CA3 region of the hippocampus, one of the projection sites of the SuM (data not shown). With these results, we attempted to characterize neurons in the SuM by *in vivo* single-neuron recording under urethane anesthesia. In this attempt, we found that the SuM had heterogeneous neuronal populations, some of which fire rhythmically at different frequency bands including theta (3–5 Hz). Among them was a previously unidentified population of high-frequency bursting SO neurons (Figure 3A), similar to cortical SO neurons characterized by up- and down-states (see STAR Methods).^17^ Cortical SO neurons fire spikes during up-states (a.k.a. active periods), whereas they remain silent during down-states (a.k.a. inactive periods).^18^^,^^19^ The SO neurons we found in the SuM exhibited a unique discharge pattern of SO which combines delta (1–3 Hz) during the down-states and rhythmic theta burst during up-states (Figure 3A, segments 1 and 2). The up-states were correlated with cortical EEG SWA (Figure 3A, segment 1). These firing features suggest that the bursting SO neurons identified in the SuM could be activated during NREM sleep and can burst rhythmically with the cortical activity during active EEG such as REM sleep. To further characterize this specific population, we selectively recorded and analyzed 92 neurons of this type from the SuM of young (*n* = 46) and aged (*n* = 46) mice. Of those, neurobiotin (Nb) labeling was applied to 22 cells, and due to the depth of the SuM and the narrow glass micropipette tip (<2 μm), only 7 neurons (*n* = 5 from 5 young mice and *n* = 2 from 2 aged mice) were successfully labeled with Nb. A majority of the Nb^+^ neurons were located in the anterior part of the SuM (Figure S3B). The Nb^+^ neurons discharged high-frequency spike-bursting SO in association with the cortical EEG SWA of young and aged mice (Figures 3A–3D). In young mice, a representative neuron (unit) of this type discharged rhythmically at ∼7 Hz during the up-states, which occurred with the large amplitude EEG SWA (Figure 3A, segment 1, asterisks). The unit also displayed a discharge pattern of delta (1–2 Hz) rhythm characterized by a clock-like spiking during the down-states (Figure 3A, segment 2, arrowheads). A single burst contained 4 spikes most frequently but occasionally 8 spikes (Figure 3A, segment 3). The unit showed short spike half-amplitude duration of ∼0.4 ms (Figure 3A, segment 4). The distribution of the unit autocorrelation histogram (ACH) and interspike interval histogram (ISIH) derived from the same unit activity are displayed (Figure 3B, left). The unit ISIH revealed high intraburst firing frequencies of 100–500 Hz (Figure 3B, middle, see STAR Methods). The ISIH also showed peak at 2.7 Hz of delta frequency. The unit displayed high firing rate (8.6 Hz, determined as the number of spikes per second) and intraburst spike count (18) within the frequency range of 100–500 Hz (Figure 3B, right).

The Nb^+^ neurons recorded in anesthetized aged mice displayed the same profile and discharge patterns as those of young mice. However, their electrophysiological features were markedly reduced as compared to those in young mice. The most pronounced changes were the decreased unit firing as evident on the representative unit activity (Figure 3C). The number of high frequency bursts during the up-states was decreased, and the number of clock-like spikes (arrowheads) occurring during the down-states were reduced as well (Figure 3C, segments 1 and 2). A single burst in the aged mice showed 2 spikes most times and rarely 4 (Figure 3C, segment 3). The spike width was not affected (Figure 3C, segment 4). The unit ACH and ISIH showed fewer total spikes compared to young mice, and delta peaks displayed lower spike counts (Figure 3D, left). The unit ISIH showed lower spike counts for intraburst spikes within the 100–500 Hz frequency range (Figure 3D, middle). The firing rate was lower (1.7 Hz) in this unit, with lower numbers of intraburst spikes, 10 at 100–500 Hz (Figure 3D, right). The means were computed from 8 neurons that showed the same discharge profiles and patterns (*n* = 5, from young mice and *n* = 3, from aged mice) (Figure 3E). This comparison clearly shows that the unit firing rate was significantly decreased in aged mice, compared with those in young mice (Figure 3E, 1). The number of intraburst spikes within the 100–500 Hz frequency range was also reduced, whereas the spike half amplitude did not differ significantly (Figure 3E, 2 and 3). We also examined the phase-locking relationship between up-states of the bursting SO neurons and EEG SWs in both young and aged mice (see STAR Methods). Polar plot analysis of these units revealed a strong phase locking to cortical EEG SO activity in both young (Figures S4A and S4B) and aged (Figures S4C and S4D) mice.

Finally, we examined whether NMN could change those unit electrophysiological features. Multiple units of the cell type described previously (*n* = 11 cells from 7 young mice; *n* = 15 cells from 6 aged mice) were first recorded during SWA under urethane before any treatment and then PBS or NMN was administered i.p. Thereafter, neurons (*n* = 28 from 4 young mice; *n* = 35 from 4 aged mice; see STAR Methods) were recorded at different time points (Figure 3F, left) during SWA. Remarkably, in aged mice, NMN administration showed a trend of progressive increase of the unit firing rate with a significant difference emerging at 2 h (Figure 3F, right top). The spike duration remained unchanged after NMN administration (Figure 3F, right bottom). We did not observe such drastic changes in young mice, although there appeared to be slight increases in the spike duration (Figure 3F, right bottom).

### The SuM bursting SO neurons are NREM/REM-active neurons, and their activity is significantly rescued by NMN in aged mice

We next examined the discharge profile and the electrophysiological property of the SuM bursting SO neurons in unanesthetized young and aged mice during the sleep-wake states. Using the same coordinates as those under anesthesia, the SuM bursting SO neurons were targeted. Only neurons that could be recorded during the three main states, NREM, REM, and wake, were selected for analysis. In young mice, the recorded units in the SuM discharged at high rates during NREM sleep, increased firing further during the transition to REM sleep (tREM), and discharged at the highest rates during REM sleep (Figure 4A). They decreased their firing during wake. The units discharged through the large amplitude SWs during NREM sleep and discharged rhythmically during REM sleep (Figure 4A). The unit ISIH displayed fast activities at 100–500 Hz and 100 Hz with its highest number of spikes during REM sleep (Figure 4B). The unit ACH depicted the theta rhythmicity during REM, peaking at 8.3 Hz (Figure 4C). These features suggest that the SuM bursting SO neurons are NREM/REM-active neurons that are most active during REM sleep.

In aged mice, whereas the discharge profile and pattern were the same as those in young mice, the unit firing and the number of intraburst spikes (100–500 Hz and 100 Hz) were reduced across the 3 main states. More importantly, the unit rhythmicity during REM and its synchrony with theta of EEG activity were disrupted as displayed for the representative unit (Figure 4D). The unit ISIH revealed multiple lower peaks (Figure 4E), and the ACH showed no apparent rhythmicity, with a very low amplitude theta peak at 7.1 Hz during REM sleep (Figure 4F).

Finally, we analyzed the electrophysiological properties of NREM/REM-active bursting SO neurons in response to saline (*n* = 3 i.p.) or NMN (*n* = 4, i.p.) administrations (Figures 4G and 4H). In saline-administered young mice, the neurons showed the highest unit firing frequency during REM sleep. There were significant differences between NREM and REM, tREM and REM, and REM and wake. As in young mice, the neurons in aged mice displayed their maximal discharge rate during REM sleep and significantly decreased during wake. Again, there were statistically significant differences between NREM and REM, NREM and wake, tREM and REM, and REM and wake. Nonetheless, the firing frequency was significantly reduced during NREM, tREM, and REM in aged mice, compared with young mice (Figure 4G, 1). The intraburst spike counts in saline-administered young and aged mice were also analyzed (Figure 4G, 2). The number of spikes of both 100–500 Hz and 100 Hz in young units showed the highest counts during REM sleep and significant decreases from REM to wake. The intraburst spike counts were significantly reduced during NREM, tREM, and REM in aged mice (Figure 4G, 2). The spike half amplitude durations were not statistically different throughout all states between young and aged mice (Figure 4G, 3).

Importantly, in NMN-administered aged mice, the unit firing frequency was significantly increased during REM sleep, compared with saline-administered aged mice (Figure 4H, 1). The intraburst spike counts of 100–500 Hz and 100 Hz also tended to increase or significantly increased, respectively, during REM sleep in NMN-administered aged mice (Figure 4H, 2). The spike half amplitude duration was not significantly altered by NMN (Figure 4H, 3). Therefore, these findings clearly demonstrate that NREM/REM-active bursting SO neurons show significant functional decline during aging, which can be rescued by the administration of NMN.

### *Slc12a8*-deficient mice exhibit significant reduction in recurrent EEG activation in response to NMN and abnormal cognitive function

**Figure 5:**
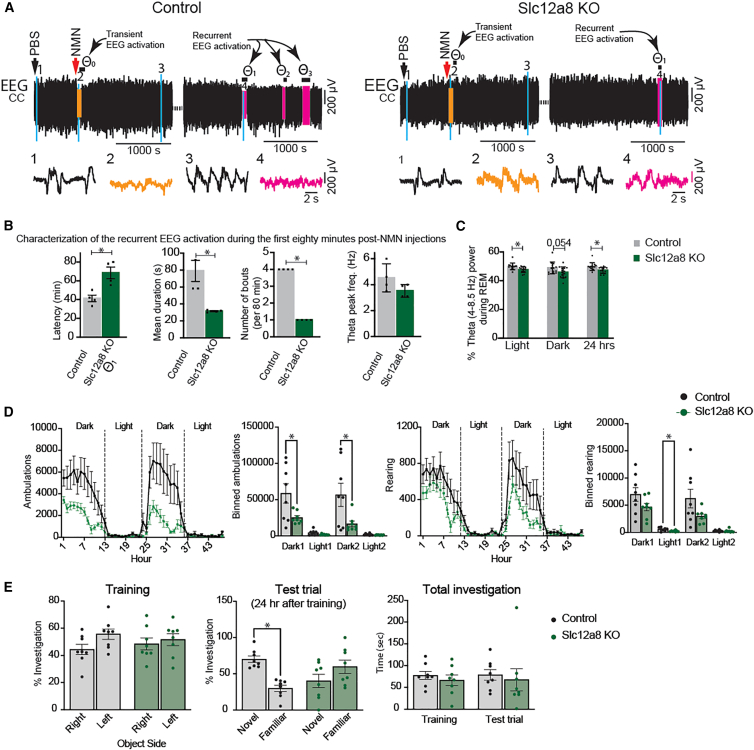
Slc12a8-deficient mice exhibit reduced recurrent EEG activation in response to NMN and abnormal cognitive function (A) EEG of wild-type control (left) and Slc12a8-KO (right) mice under urethane anesthesia are shown. Segments 1–4 on EEG traces are expanded below. (B) The latency, duration, bouts, and frequency (3–6Hz) peak power of recurrent EEG activation under urethane anesthesia are shown (∗*p* < 0.05, unpaired *t* test, *n* = 4 per group). (C) Percent theta power (4–8.5 Hz) during REM sleep across 24 h in freely moving control and Slc12a8-KO mice (∗*p* < 0.05, Student’s *t* test, *n* = 13–14 per group). (D and E) Behavioral tests in Slc12a8-KO and wild-type littermate control female mice. (D) Ambulations (left) and rearings (right) measured over 48 h. Light and dark periods are indicated (∗*p* < 0.05, two-way repeated measures ANOVA with uncorrected Fisher’s LSD post hoc test, *n* = 8 mice each group). (E) Novel object recognition was assessed. Percent object investigation times during training (left) and a test trial conducted 24 h later (middle), and total object investigation times (right) are shown (∗*p* < 0.05, two-way repeated measures ANOVA followed by a two-stage linear step-up procedure of Benjamini, Krieger, and Yekutieli to correct for multiple comparisons by controlling the FDR, *n* = 8 mice each group). Results are presented as mean ± SEM.

NMN is transported into cells and tissues through an NMN transporter Slc12a8.^20^^,^^21^ Thus, we suspected that *Slc12a8*-deficient mice would not exhibit recurrent EEG activation in response to NMN. To address this possibility, we compared the response to NMN between wild-type control and *Slc12a8*-knockout (*Slc12a8*-KO) mice under urethane anesthesia. As shown in Figure 1, NMN induced a transient EEG activation in all wild-type control mice but only in a half of the *Slc12a8*-KO mice (Figure 5A, segment 2, left and right). The durations of this transient EEG activation were 78.7 ± 6.6 s and 61.5 ± 11.5 s in control and *Slc12a8*-KO mice, respectively. More importantly, *Slc12a8*-KO mice exhibited significant changes in the latency, duration, and number of bouts of recurrent EEG activation. The latency of recurrent EEG activation was significantly increased in *Slc12a8*-KO compared with control mice, whereas the duration and number of bouts of recurrent EEG activation were significantly decreased in *Slc12a8*-KO mice (Figure 5B). Additionally, the theta (3–6 Hz) frequency peak during recurrent EEG activation tended to be decreased in *Slc12a8*-KO mice (Figure 5B, right). It has been reported that the attenuation of the theta rhythm during REM sleep suppresses novel object recognition.^22^ Thus, we examined both low theta (4–8.5 Hz) and high theta (6–10 Hz) power during 24 h as these oscillations have distinct sensitivities to certain manipulation^23^^,^^24^ and conducted novel object recognition test in freely moving control and *Slc12a8*-KO mice. Interestingly, only the percent theta (4–8.5 Hz) power was significantly decreased through a 24-h period in *Slc12a8*-KO compared to control mice (Figure 5C), whereas other sleep parameters did not show significant differences (Figure S5). Furthermore, *Slc12a8*-KO mice showed significantly decreased locomotor activity and a trend of decreased rearing during the dark time, compared to control mice (Figure 5D). Importantly, *Slc12a8*-KO mice did not show normal preference to a novel object over a familiar object 24 h after training, whereas control mice properly showed more investigation on a novel object (Figure 5E, middle). Control and *Slc12a8*-KO mice did not show any difference in their training, and they also had similar total investigation times in both training and test trials (Figure 5E, left and right). These results provide evidence for the notion that direct uptake of NMN through Slc12a8 is critical to induce REM sleep and also maintain normal cognitive function in mice.

### Photostimulation of SuM GABAergic neurons induces SO and theta rhythm in the cortical EEG

**Figure 6:**
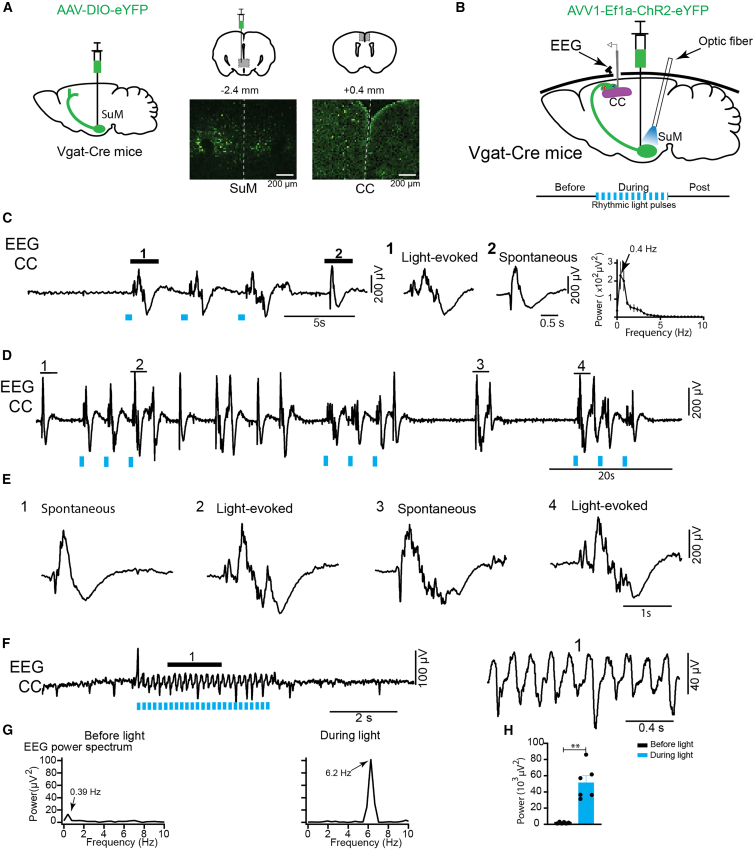
Rhythmic light pulse stimulation to the SuM GABAergic neurons induces SO and theta rhythm in the CC under urethane anesthesia (A) Scheme of the brain showing viral injection into the SuM of *Vgat*-Cre mice for anterograde tracing, and the cingulate cortex (CC) as one of the projection sites for the SuM GABAergic neurons in Vgat-Cre mice. Each scale bar represents 200 μm. (B) Scheme showing viral injection to the SuM- and EEG-recording site, and single unit and optic fiber position in Vgat-Cre mice. (C) A train of three pulses (500 ms-ON, 0.2 Hz) was applied during an inactive (down-states) period of EEG that yielded SO resembling to spontaneous cortical ones. Segments (1 and 2) of light evoked response and spontaneous slow waves of EEG are expanded, and mean EEG power spectrum is shown at right. Note the slow activity peak at 0.4 Hz (*n* = 3 mice). (D and E) Multiple trains of rhythmic light pulse stimulation were applied at different time intervals to evoke SO. Segments (1–4) on EEG are expanded in (E). (F) Left, rhythmic light pulse (pulse duration 200 ms at 6 Hz) stimulation induced rhythmic theta activity. Right, the segment (1) on EEG is expanded. (G) EEG power spectra peaks at slow 0.39 Hz before and at 6.2 Hz (theta frequency) during light are shown by arrows. (H) EEG theta power showed significant increases during light in comparison with before light stimulation (*n* = 6 mice, ∗∗*p* < 0.01, paired, two-tailed Student’s *t* test). Results are presented as mean ± SEM.

Interestingly, it has been reported that the SuM has many neurons that are both glutamatergic and GABAergic.^25^^,^^26^ A recent study demonstrates that the SuM glutamatergic neurons are key to promote wakefulness in the SuM.^26^ Given that NMN enhances REM sleep (see Figure 2A), we presumed that NMN might work through GABAergic neurons in the SuM. Thus, we first examined whether the SuM GABAergic neurons express Slc12a8. We conducted triple RNAscope *in situ* hybridization for *Vglut2*, *Vgat*, and *Slc12a8* on brain sections covering the SuM. Surprisingly, throughout the SuM, 42%–50% of *Slc12a8*-positive neurons are glutamatergic/GABAergic double positive (Figure S6A). If NMN primarily stimulates the GABAergic activity of those neurons, we should be able to recapitulate the induction of SO and theta oscillations in the cortex by optogenetic stimulation of the SuM GABAergic neurons. We were able to confirm that the SuM GABAergic neurons had projections to the CC (Figure 6A), which is consistent with the EEG recording site throughout the present study, as well as to the septum, claustrum, and hippocampus (Figure S6B). Using *Vgat*-Cre mice injected with the double-floxed ChR2-eYFP adeno-associated virus (AAV) into the SuM, as previously described,^27^ we conducted optogenetics to specifically target the SuM GABAergic neurons by selectively expressing ChR2 in those neurons in *Vgat*-Cre mice (see STAR Methods, Figure 6B). Remarkably, rhythmic light pulses (500 ms-On, <1 Hz) mimicking SO pattern delivered to the SuM triggered SO that resembled to spontaneous ones in the CC (Figure 6C). The power spectrum revealed a peak during photostimulation at 0.4 Hz of SO (Figure 6C, right). Multiple trains of the same rhythmic light pulses were delivered to the SuM, and every single one elicited SO activity (Figure 6D), resembling to spontaneous activity (Figure 6E). By filtering EEG, both light-evoked slow waves and spontaneous ones were found to have all large slow wave with delta, spindles, and gamma frequencies nested on it (Figure S7). Because theta oscillation (3–6 Hz) is the prominent rhythm of recurrent EEG activation under urethane (Figure 1) and during REM sleep, we next examined whether the theta rhythm could be induced in cortical EEG. A rhythmic pulse (200 ms, 4 or 6 Hz) at a theta frequency band delivered to the SuM evoked theta rhythm in cortical EEG (Figures 6F, S7E, and S7F). The EEG spectral power showed an evident peak at 6.2 Hz of theta frequency during photostimulation (Figure 6G). The EEG power was significantly enhanced during photostimulation, compared with that before stimulation (Figure 6H). These results suggest that the activation of SuM GABAergic neurons that express Slc12a8 can trigger SO and theta rhythm in the cortex.

### Photostimulation of SuM GABAergic neurons or their terminals in the CC reduces the firing frequency of cortical neurons

**Figure 7:**
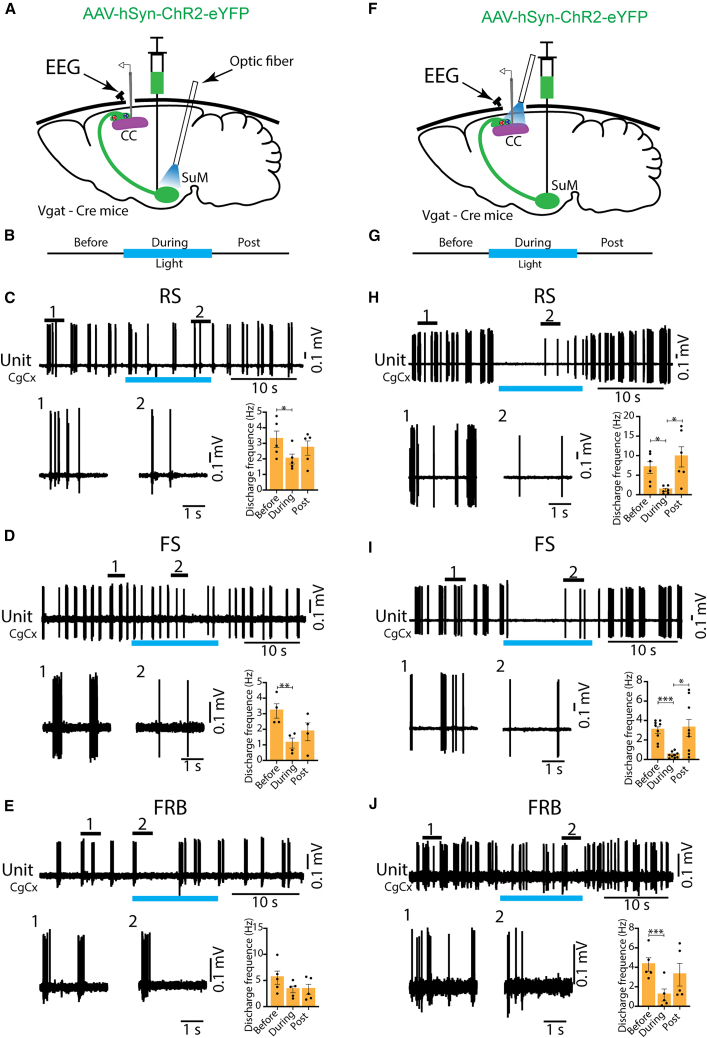
Continuous light pulse to the SuM GABAergic neurons or their axonal terminals in the CC reduces cortical neurons firing frequency under urethane anesthesia (A–J) Scheme of the brains showing viral injection sites and optogenetic fiber stimulation to the SuM (A) or axonal terminals in CC (F). Schematic of optogenetic stimulation protocol for the SuM (B) and the axonal terminals (G). Segments 1 and 2 on the units of RS neurons are expanded below with plots showing significant decrease of units discharge frequency during continuous light pulse stimulation (C, *n* = 5, from 4 mice; H, *n* = 6, from 3 mice, ∗*p* < 0.05, One-way ANOVA with Tukey post hoc test). Units of FS neurons showed significantly decreased firing frequency during continuous light stimulation (D, *n* = 4, from 4 mice, ∗∗*p* < 0.01; I, *n* = 9 from 3 mice, ∗*p* < 0.05, ∗∗∗*p* < 0.001, One-way ANOVA with Tukey post hoc test). Units of FRB neurons showed a trend of decreased firing frequency during SuM stimulation (E, *n* = 5 from 3 mice) and significantly decreased firing during axonal terminal stimulation (J, *n* = 5 from 3 mice, ∗∗∗*p* < 0.001, One-way ANOVA with Tukey post hoc test). Results are presented as mean ± SEM.

We next assessed the effect of optogenetic stimulation of the SuM GABAergic neurons or their axonal terminals on cortical neurons under urethane anesthesia. Previous studies have demonstrated that cortical pyramidal neurons, a.k.a regular spiking (RS) neurons, reduce their firing rates during sleep.^28^ Wide-field calcium imaging studies further revealed that SW sleep is associated with a global decrease in pyramidal cell activity, and this reduction in network activity becomes even more pronounced during REM sleep.^29^ Therefore, we hypothesized that optogenetic activation of SuM GABAergic neurons (Figures 7A and 7B) or their axonal terminals within the CC (Figures 7F and 7G) reduces the firing of cortical neurons. Using *Vgat*-Cre mice expressing ChR2 described previously, unit activities of cortical SO neurons were recorded in the CC and classified based upon their spike half amplitude duration (ms) and firing train^30^^,^^31^: RS (0.60 ± 0.07 ms, *n* = 5), fast spiking (FS, 0.36 ± 0.03 ms, *n* = 4), and fast-rhythmic bursting (FRB, 0.31 ± 0.02 ms, *n* = 5). Continuous light stimulation to the SuM significantly reduced the firing frequency of both RS and FS neurons, with a trend of moderately decreased activities of FRB neurons (Figures 7C–7E). Similarly, continuous light stimulation to axonal terminals of SuM GABAergic neurons within the CC also reduced the firing frequency of RS, FS, and FRB neurons in the CC (Figures 7H–7J). These results suggest that the SuM GABAergic neurons and their axonal terminals within the CC can reduce the firing frequency of cortical neurons, most likely facilitating NREM and REM sleep during regular sleep-wake cycles.

### Chemogenetic suppression of SuM GABAergic neurons decreases REM sleep in freely moving young mice

**Figure 8:**
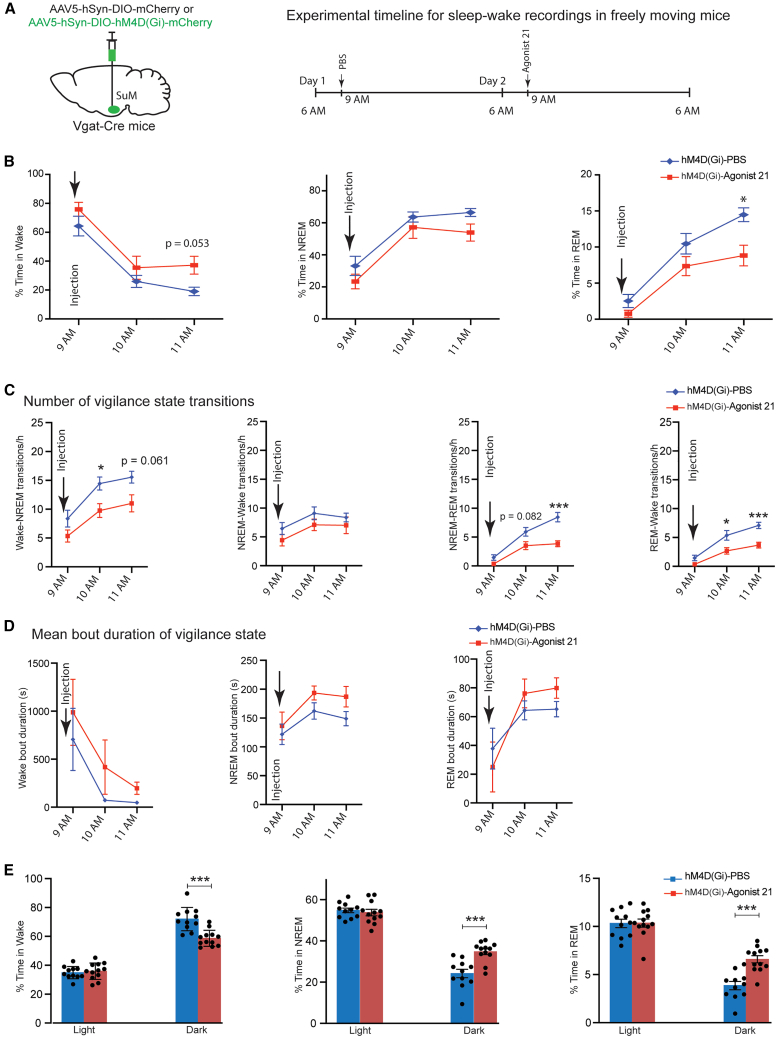
DREADD inhibition of the SuM GABAergic neurons increases light-phase REM and drives dark-phase NREM/REM rebound (A) Scheme of viral injection into the SuM and experiment protocol (*n* = 12 mice per group). (B) The percentages of time spent in wake, NREM, and REM (*n* = 12 mice per group, ∗*p* < 0.05, two-way ANOVA with Šidák post hoc test. (C) Number of vigilance state transitions (∗*p* < 0.05, ∗∗∗*p* < 0.001, two-way ANOVA with Šidák post hoc test). (D) Mean bout duration of vigilance states during wake, NREM, and REM sleep. (E) The percentages of time spent in wake, NREM, and REM during light and dark phases (∗∗∗*p* < 0.001, paired, two-tailed Student’s *t* test). Results are presented as mean ± SEM.

Lastly, by knocking down *Slc12a8* specifically in the SuM, we attempted to prove the direct connection between the NMN/Slc12a8 system and NREM/REM sleep regulation in the SuM. Unfortunately, the knockdown efficiency for *Slc12a8* was minimal in the SuM, and therefore, we had to take an alternative approach. We decided to employ a chemogenetic technique known as designer receptors exclusively activated by designer drugs (DREADDs) to selectively inhibit the activity of the SuM GABAergic neurons. An AAV carrying the double-floxed hM4D(Gi) or control mCherry construct was injected stereotactically into the SuM of *Vgat*-Cre mice (Figure 8A, left). We administered Agonist 21 (1 mg/kg, i.p.), a potent and selective agonist of hM4D(Gi),^32^ at 9 a.m. to inhibit the SuM GABAergic neurons expressing hM4D(Gi) in freely moving young mice, and EEG and EMG activities were recorded for two consecutive days (Figure 8A, right, see STAR Methods). Agonist 21-administered mice showed a trend of increase in percent time in wake, compared with PBS treatment in the same individuals, over 2 h post-administration (Figure 8B, left). Percent time in NREM sleep showed a trend of slight decreases in Agonist 21-administered mice, compared with PBS-treated mice (Figure 8B, middle). Remarkably, percent time in REM sleep was significantly suppressed at 11 a.m. time point in Agonist 21-treated mice, compared with PBS-treated mice (Figure 8B, right). Consistent with those results, the number of vigilance state transitions in Agonist 21-administered mice showed reduced transition frequencies in wake-NREM, NREM-REM, and REM-wake, compared with PBS-treated mice (Figure 8C). The mean bout duration of all vigilance states in Agonist 21-administered mice did not differ significantly from those in PBS-treated controls (Figure 8D). Interestingly, during the dark time, Agonist 21-treated mice showed reciprocal responses, namely, a significant decrease in percent time in wake and significant increases in percent time in both NREM and REM sleep, compared with PBS-treated mice (Figure 8E). Vigilance state transitions also exhibited significant increases in wake-NREM, NREM-wake, NREM-REM, and REM-wake (Figure S8A). Additionally, the mean wake bout duration was reduced in Agonist 21-administered mice during the dark phase (Figure S8B). mCherry-expressing control mice treated with Agonist 21 showed no significant changes in any of their sleep profile (Figures S8C and S8D). All these results from the DREADDs experiment provide strong evidence supporting that the SuM GABAergic neurons, which express *Slc12a8*, play an important role in promoting REM sleep.

## Discussion

In this study, we have identified a previously uncharacterized population of bursting SO neurons in the SuM that respond to NMN, an important NAD^+^ intermediate. These neurons have a unique discharge pattern, characterized by rhythmically bursting during the up-states and delta rhythm during the down-states of SO. The analysis of those bursting SO neurons in an unanesthetized condition revealed that they are NREM/REM-active neurons that display the highest activity during REM sleep. Indeed, NMN induces transient and recurrent EEG activations in urethane-anesthetized mice and also enhances REM sleep in unanesthetized, head-fixed mice. Importantly, these NREM/REM-active, bursting SO neurons showed significant functional decline during aging, whereas their firing frequency was significantly restored by NMN in aged mice. Additionally, *Slc12a8*-KO mice showed a significant defect in response to NMN, decreased percent theta power, and impaired novel object recognition, suggesting that NMN plays a critical role in the maintenance of normal REM sleep and cognitive function. Because *Slc12a8* is expressed in glutamatergic/GABAergic double-positive neurons in the SuM and also because optogenetic stimulation of the SuM GABAergic neurons induced SO and theta rhythm in the CC, it is conceivable that NMN most likely stimulates the GABAergic, *Slc12a8*-expressing, bursting SO neurons in the SuM and enhances REM sleep. More importantly, DREADD-mediated inhibition of the SuM GABAergic neurons induced a significant decrease in REM sleep during the light time in freely moving young mice, providing strong support to the importance of those neurons in promoting REM sleep.

In the rat SuM, bursting neurons have been shown to discharge rhythmically in phase with hippocampal theta.^33^ Indeed, neurons in the SuM project to the hippocampus^25^ and cortex^26^ and can exert a significant modulatory control of the theta rhythm^34^ originated in the hippocampus.^35^ Most recently, it has been demonstrated that the SuM neurons projecting to the hippocampal CA2 region are highly active during REM sleep but quiet during NREM/wake and specifically required for the consolidation of social memory.^36^ Because the bursting SO neurons that we identified in this study are NREM/REM-active, the SuM neurons reported to project to the hippocampal CA2 most likely comprises a different neuronal population. Nonetheless, it will be of great importance to further characterize these SuM neurons that contribute to the regulation of REM sleep. A critical finding in this study is that the NREM/REM-active, bursting SO neurons are significantly altered during aging. The unit discharge frequency, theta during the up-states and delta rhythmicity during the down-states are disrupted in aged mice. Remarkably, NMN can restore the unit discharge frequency in aged mice, strongly suggesting that NAD^+^ decrease may cause such functional decline in the SuM. Given that *Slc12a8* deficiency reduces tissue NAD^+^ levels^20^ and causes defects in NMN response, theta power, and novel object recognition in mice, one interesting possibility is that the supply of NMN to those neurons may decrease during aging. However, why NMN has different effects on NREM and REM sleep between young and aged mice still remains a big question. In particular, NMN can enhance NREM sleep as well as REM sleep in young mice, whereas it promotes only REM sleep in aged mice. The regulation of NREM sleep requires a complex circuit involving multiple interconnected regions, including the preoptic area, brainstem, hypothalamus, and other regions.^37^^,^^38^ Therefore, it is possible that there are other subsets of neurons that are involved in such an NREM sleep-regulatory circuit and respond to NMN in young mice but not in aged mice. Given that the chemogenetic inhibition of the SuM GABAergic neurons predominantly decreases REM sleep, the projection of the SuM GABAergic neurons to the CC may comprise an important part of the REM sleep-regulatory circuit, which is sensitive to the availability of NMN during aging. This idea also provides a reasonable explanation for why NMN can enhance the unit firing activity only in aged mice.

In conclusion, our findings in this study open an interesting opportunity to further explore a potential of NMN as an effective candidate for therapeutics that can prevent or treat age-associated REM sleep problems in the elderly. Further investigation will be necessary to elucidate the molecular mechanism by which NMN activates the bursting SO neurons and how exactly aging affects the mechanism in those neurons.

### Limitations of the study

In our present study, we primarily used urethane anesthetization that allowed us to conduct juxtacellular recording in the SuM. Whereas this experimental approach led us to the discovery of the SuM bursting SO neurons, molecular features of this specific subset of neurons remain unknown, and therefore, we were not able to genetically manipulate these neurons and conduct sleep analysis in freely moving mice. Additionally, due to the lack of SuM-specific *Slc12a8* knockdown, whether NMN directly stimulates *Slc12a8*-expressing GABAergic neurons in the SuM to influence REM sleep remains unresolved. Because *Slc12a8* KO mice show normal sleep architecture, further detailed investigation of the SuM *Slc12a8*-expressing, bursting SO neurons, and the role of NMN in natural sleep will be required, and genetic manipulation of their neuronal activity will provide additional support to our conclusion in the near future.

## Resource availability

### Lead contact

Further information and requests for resources and reagents should be directed to the lead contact, Shin-ichiro Imai (imaishin@wustl.edu).

### Materials availability

The *Slc12a8*-KO mice were generated as described previously^20^ and are available upon request. Other mice were obtained from Charles River Laboratories and Jackson Laboratories as listed in the key resources table. All AAVs were obtained from Addgene.

### Data and code availability

This paper does not report original code. All data generated or analyzed during this study are included in this manuscript. Any additional materials are available from the corresponding author on reasonable request.

## Acknowledgments

We thank C. Ito and Y. Koike for their technical assistance on immunohistochemistry and S. Johnson and Y. Nishiguchi for the long-term NMN administration. We also thank members of the Imai lab for valuable discussions. This work was mainly supported by grants to S.I. from the 10.13039/100009619Japan Agency for Medical Research and Development (AMED), Tokyo, Japan, 17∼21gm501002, “The Project for Elucidating and Controlling Mechanisms of Aging and Longevity”, the 10.13039/100000049National Institute on Aging (AG037457 and AG047902), and 10.13039/100011912Washington University School of Medicine Tanaka Fund. This work was also supported by a grant to the 10.13039/100020432Intellectual and Developmental Disabilities Research Center at Washington University from the 10.13039/100009633Eunice Kennedy Shriver National Institute of Child Health & Human Development (P50 HD103525), by the Animal Behavior Core supported in part by the 10.13039/100009607McDonnell Center for Systems Neuroscience and the Taylor Family Institute, and by the Hope Center Animal Surgery Core.

## Author contributions

S.I. and Y.C. conceived and supervised the project, and Y.C., C.S.B., and S.I. mainly designed research. Y.C. performed all electrophysiology experiments, C.S.B conducted c-Fos immunostaining and triple RNAscope *in situ* hybridization, and C.S.B. and V.H. conducted the anterograde tracing experiment. C.S.B. and Y.C. collaborated with C.M.Y. to conduct novel object recognition test and also with N.R. and M.W. to examine theta power during 24 h. Y.C., C.S.B., V.H., C.M.Y., N.R., M.W., and S.I. were all involved in data analysis and interpretation. Y.C. and S. I. mainly wrote the manuscript, and other authors reviewed and edited the manuscript.

## Declaration of interests

S.I. receives a part of patent-licensing fees from MetroBiotech (USA) and the Institute for Research on Productive Aging (Japan) through Washington University. S.I. also serves as the President of the Institute for Research on Productive Aging (Japan) and a co-CEO of LongGen Bioscience. S.I.’s External Professional Activities (EPAs) have already been reported to and a potential conflict of interest has been properly resolved through the Washington University Conflict of Interest Committee.

## Declaration of generative AI and AI-assisted technologies in the writing process

The authors used neither generative AI nor AI-assisted technologies during the preparation of this work.

## STAR★Methods

### Key resources table

**Table:** 

REAGENT or RESOURCE	SOURCE	IDENTIFIER
**Antibodies**		

Rabbit anti-cFos	Synaptic Systems	226003; RRID: AB_2231974
Chicken anti-GFP	Abcam	ab13970; RRID: AB_300798
Alexa Fluor 647 goat anti-rabbit	Jackson Immunoresearch	111-605-045; **RRID:**AB_2338075
DyLight 488 goat anti-chicken IgY	Invitrogen	SA5-10070; RRID: AB_2556650
Cy3 Streptavidin	Jackson Immunoresearch	**016-160-084; RRID:**AB_2337244

**Bacterial and virus strains**		

AAV5-Ef1a-DIO-EYFP	Karl Deisseroth	Addgene; 27056- AAV5
AAV1-Ef1a-DIO-hChR2(H134R)-eYFP-WPRE	Karl Deisseroth	Addgene; 20298-AAV1
AAV5-hSyn-DIO-mCherry	Bryan Roth	Addgene; 50459- AAV5
AAV5-hSyn-DIO-hM4D(Gi)-mCherry	Bryan Roth	Addgene; 44362- AAV5

**Chemicals, peptides, and recombinant proteins**		

Urethane	Sigma-Aldrich	U2500-100G
Neurobiotin	Vector Laboratories	SP-1120
NMN	Mirailab Bioscience	N/A
Paraformaldehyde (16%)	Electron Microscopy Sciences	15710
DAPI	Sigma-Aldrich	D9542
Triton-X	Sigma-Aldrich	T8787
DREADD Agonist 21	Hello Bio	HB6124

**Critical commercial assays**		

RNAscope Multiplex Fluorescent	Advanced Cell	323100
Reagent Kit v2	Diagnostics Bio	
TSA Plus Fluorescein	Akoya Biosciences	NEL741001KT
TSA Plus Cyanine 3	Akoya Biosciences	NEL744001KT
TSA Plus Cyanine 5	Akoya Biosciences	NEL745001KT

**Experimental models: Organisms/strains**		

Mouse: C57BL/6J	Charles River Laboratories (Japan)	N/A
Mouse: Slc12a8-KO	Grozio et al.^22^	N/A
Mouse: Vgat-Cre: *Slc32a1*^*tm2(cre)Lowl*^/J	Jackson Laboratories	016962

**Oligonucleotides**		

RNAscope Probe mm-Slc32a1 (*vGlut2)*	Advanced Cell Diagnostics Bio	319191
RNAscope Probe mm-Slc17a6 (*vGAT)* Diagnostics Bio	Advanced Cell	1170921
RNAscope Probe mm-Slc12a8	Advanced Cell Diagnostics Bio	846731

**Software and algorithms**		

Spike Histogram	ADInstruments	MLS062/8
Peak Analysis Module	ADInstruments	MLS380/8
Spectrum analysis	ADInstruments	MLS060/8
Unit-EEG phase locking analysis	MathWorks	https://www.mathworks.com
Igor Pro	Wavemetrics	https://wavemetric.com
Image J	NIH	https://imagej.nih.gov/ij
Prism 9	Graphpad	https://www.graphpad.com
CellProfiler	Broad Institute	https://cellprofiler.org

**Other**		

Superfrost Plus Slides	Thermo Fisher	12-550-15
Goat serum	Vector Laboratories	S-1000

### Experimental model and study participant details

All animal studies were reviewed and approved by the Institutional Animal Ethics Committees at RIKEN-BDR and Institute of Biomedical Research and Innovation in the Foundation for Biomedical Research and Innovation, Kobe, Japan (Figures 1, 2, 3, 4, S1, and S2), and the Washington University Institutional Animal Care and Use Committee in St. Louis, Missouri, USA (Protocol No. 22-0007) (Figures 5, 6, and S2–S5), and performed following the RIKEN-BDR and NIH guidelines. Recordings were performed on young (3-5 month old) and aged (20-24 month old) wild type C57BL/6J male mice purchased from Charles River Laboratories (Japan). Mice were housed under a 12:12 h light/dark cycle and had free access to food and water. Experimental paradigms included short-term recording under urethane anesthesia or long-term recording in unanesthetized head-fixed or freely moving systems following stereotaxic implantation of electrodes for EEG recording. *Slc12a8*-knockout (*Slc12a*8-KO) mice^20^ and wild type littermate controls were bred and maintained at Washington University in St. Louis. Both male and female mice at 4-6 months of age were used for freely moving EEG/EMG recording. Female mice at 12-15 months of age were used for behavior testing. *Vgat*-Cre mice (#016962, Jackson Laboratories) were bred and maintained at Washington University.

### Method details

#### Electrophysiological recordings

For short-term recording, a mouse was placed in a Plexiglass box, and anesthesia was induced with isoflurane at ∼5%. The anesthetized mouse was positioned on a stereotaxic frame (David Kopf Instruments) including a heating pad to maintain body temperature at 36-37°C for the duration of surgery. Isoflurane was maintained at ∼2.5% through a mask, and body temperature was confirmed through a rectal thermal probe throughout the surgery. To record the electroencephalogram (EEG), stainless steel screws were placed bilaterally over the cingulate cortex at the coordinate [anteroposterior (AP): 0.3 mm and mediolateral (ML): 0.5 mm] relative to bregma and unilaterally in the frontal bone as a reference. The isoflurane concentration was lowered and replaced by urethane (ethyl carbamate, Sigma) at a large dose (1 g/kg, i.p.) as the first dose to start the recording. The animal in the stereotactic frame was then transferred to the recording chamber, and EEG was recorded. During the recording, additional small doses of urethane (0.1-0.15g/kg) were given as necessary if mice responded to pinching of the hind limb. To record the unit activity under urethane anesthesia, two holes were drilled in the skull at the stereotactic coordinates: AP:1.3 to -2.7 mm; ML: 0.0 to 0.5 mm for the glass micropipette insertion. The micropipette was inserted at AP: -2.2 to -2.5 mm; ML: 0.2 to 0.34 mm; dorsoventral (DV): 4.2 to 5.3 mm. The dura mater was opened in each hole before starting the recording.

For long-term recording, stereotactic surgery was performed on animals to implant the electrodes and a head-fixation post. Anesthesia was induced (5%) and maintained with isoflurane. The screws for EEG were implanted as described above, and silver wire loops were inserted in the neck muscles for electromyogram (EMG) recording. The EEG and EMG wires were grouped tightly and sent to a connector fixed to the left side of the head. Acrylic cement was applied to all the screws, grouped wires and head post to hold up the connector. A clean skull was left exposed for subsequent opening to insert the micropipette at the time of recording (as above). Mice were recovered from surgery for 5 days. Following recovery, the mice began to habituate to stay lying in the head fixation apparatus. For head fixation, the implanted head post was inserted into a receptable head-key on the head-fixation apparatus, and mice laid in a tube lined with foam rubber. Animals were habituated to head fixation during repetitive sessions by increasing daily habituation time from ∼ 30 min up to 5 h over a period of 6-14 days until the mice stayed comfortably awake or asleep during a full session over the afternoon recording period (light time). After completing habituation, on the day before unit recording, the mice underwent stereotactic surgery to open two holes over the SuM (see coordinates above) for the micropipette insertion. Following the short surgery (∼30 min), the mouse was taken back to the home cage for the night. All the procedures have been previously reported.^39^

#### EEG and EMG recording in mice

Custom wire EEG/EMG electrode sets were constructed using six Teflon coated stainless steel wires (76 mm bare diameter) soldered to an electronic pin header. For EEG, a screw was attached to the opposite end of the wire, and for EMG electrodes, the Teflon coating was removed approximately 2 mm from the end of the wire. The soldered contacts were covered with dental cement, and the electrode set was sterilized for implantation.

Mice were placed under 3-4% isoflurane on a stereotactic frame with a heating pad set to 36.5°C until pedal withdraw reflex ceased. The skin was prepared with betadine and alcohol wipes, with isoflurane maintained at 1-1.5% for the remainder of the procedure. After a midline vertical incision to expose the skull, forceps and 3% hydrogen peroxide were used to remove any connective tissue and dry the skull for electrode placement. Burr holes for the frontal reference electrode were made (anterior +0.8mm, lateral 0.5mm; bregma) using a micro drill with a 0.7mm tip, and screws were secured in the skull. Two bilateral active recording electrodes were placed over the parietal cortex (posterior -2.5mm, lateral ± 1.5; bregma), and a ground screw was secured over the cerebellum (posterior -6.4mm, lateral ± 0.5; bregma) using the same technique as the reference electrode. The exposed end of the two wire electrodes were inserted in the neck muscle for nuchal EMG recordings with the coated portion of the wire bent to follow the contour of the head. The exposed skull and all wires were covered in a layer of dental cement (SNAP, Parkell) with the pin header secured to the head for subsequent recording. The skin was sutured around the exposed dental cement/pin header, and tissue glue (Vetbond, 3M) was used to close the remainder of the incision. Mice received Buprenorphine (0.1mg/kg) and recovered in a warmed chamber for two hours and then recovered in recording cages.

For EEG monitoring, groups of four mice (2 control; 2 Slc12a8-KO) in individual caging recovered from surgery at least 72 hrs prior to connection with a custom flexible cable attached to the exposed pin header for recording. Freely moving but tethered mice were then acclimated to the recording cage for two weeks with normal 12hr light/dark cycles. Bilateral cortical EEG signals were acquired using a referential montage with Grass P511 AC amplifiers and BioPac MP150 DAQ and acquisition software. Signals were amplified at 10,000X with high-pass (0.5Hz) and low-pass (100Hz) filters applied. EMG signals were filtered with high-pass (10Hz) and low-pass (300Hz) filters. EEG and EMG signals were digitized at 250Hz and collected in 24-hr sessions.

To score vigilance states, bilateral EEG and nuchal muscle EMG files were imported into the LabChart software (AD Instruments), and a digital bandpass (1-70Hz EEG; 10-100Hz EMG) filter applied for review. The vigilance states of mice were manually scored in 10 second epochs as awake, NREM sleep or REM sleep, using a combination of the EEG, EMG and respective spectral power representations. Vigilance state scoring parameters were defined based on standard criteria for adult rodents. Wakefulness was defined as periods of cyclic lower amplitude mixed frequency EEG and high tone muscle activity EMG for greater than half of the epoch duration. Brief arousal periods with 5-10 sec of high muscle tone between sleep transitions were also scored as awake. EEG periods dominated by higher amplitude delta wave activity with nuchal muscle atonia were scored as NREM sleep epochs. REM sleep consisted of periods of semi-uniform theta activity EEG with muscle atonia and/or muscle atonia with brief myoclonic twitches. Genotypes and conditions were blinded during the experimental procedures.

For sleep analysis, time spent in each vigilance state was tabulated using LabChart and calculated using the scored comment for each epoch and averaged per hour. Total time spent in vigilance state, number of vigilance state transitions, and mean bout durations were averaged for each 12 hours light/dark cycle. The percentage of total power in theta range (4-8.5Hz) were calculated using the fast Fourier transformation constructed in LabChart with a 512 bin size and a Hann (cosine-bell) data window with spectral data extracted from each vigilance epoch and averaged per hour. Epochs with obvious movement artifact or epochs with vigilance state specific artifact thresholds were excluded from spectral analysis.

#### *In vivo* single neuron recording

To record the unit activity, the next day after habituation, mice were placed in the head-fixation apparatus with the head post fixed within the stereotactic frame. The open skull was perfused with lidocaine (∼15 min), washed up with saline, and the dura was opened to allow the insertion of the glass pipette. The mouse was then transferred to the recording chamber, and the micropipette mounted on a micropositioner (David Kopf Instrument, model 2660) was lowered vertically to the SuM. The micropipette (∼1 mm tip) was filled with ∼5% Neurobiotin (Nb; Vector Laboratories) in 0.5 M NaCl solution. A high input impedance DC amplifier (Neurodata IR-283A, Cygnus Technology) was used to record the single units and for juxtacellular labeling of the neuron. The unit signal was amplified (1000x, FL-01, Cygnus Technology), digitized at a sampling rate of 20 kHz, and filtered (bandpass filter: 300-3,000 Hz). The unit was simultaneously recorded with the EEG (sampled at 500 Hz and filtered between 0.5-100 Hz) and EMG (filtered between 0.5-100 Hz) with EEG amplifier (16 Channel amplifier, model 3500, A-M systems Inc.). All the signals were acquired and monitored online by LabChart v8 (ADinstruments). After obtaining the unit activities of our target cells and recording those cells during at least one full sleep-wake cycle, positive current pulses (1-10 nA, 200 ms) were delivered for a period ∼10 min to label the cells with Neurobiotin (Nb).^39^ Although multiple units were recorded from every mouse, only one cell per brain hemisphere was juxtacellularly labeled with Nb.

At the end of the recording, mice were administered an overdose of urethane (5 g/kg, i.p.) and perfused transcardially with 20 ml of cold PBS, followed by 20 ml of cold 2% paraformaldehyde solution for fixation of the brain. The brains were removed, post-fixed overnight at 4°C in the fixative solution and immersed for 1-2 d in 30% sucrose in phosphate buffer for cryoprotection and then frozen at -80°C.

#### Electrophysiological analysis

The unit mean discharge frequency and spike half amplitude duration were used to determine the physiological features of single unit (Figures 3 and 4). SO neurons are of cortical origin and exhibit activity in the 0.1-1 Hz range, characterized by alternating Up- and Down-states. During the Up-states, these neurons show sustained depolarization and repetitive spiking, which is phase-locked to the positive deflection of EEG slow waves (see Figure S4). In contrast, during the Down-states, they are hyperpolarized and largely silent.^17^ In addition, the unit autocorrelation histogram (ACH) and interspike interval histogram were computed to determine rhythmic activity in the cell and to capture the instantaneous frequency, respectively. The ACH and ISIH were computed during anesthesia and the three major states, NREM sleep, REM sleep, and wake (active wake), and EEG power spectra were analyzed. Within the bursting events, each of the events discharged intraburst spikes at broad high frequencies, which we classified to two distinct bands, 100 Hz and 100-500 Hz, based on previous studies.^40^^,^^41^

To assess the effects of PBS or NMN on neuronal firing (Figure 3F), recordings were conducted in young (n=4) and aged (n=4) mice. For each mouse, unit recordings were performed before drug injection (baseline) and at three post-injection time points: 30 minutes, 1-h, and 2- hr time points. 1) If only one neuron was recorded for a given mouse at a given time point, the firing rate of that neuron was used directly; 2) If multiple neurons were recorded in the same mouse at a given time point, the average firing rate of all recorded neurons was calculated and used as the representative value for that time point in that mouse; 3) This approach was applied consistently across all time points (baseline, 0.5-hr, 1-hr, and 2-hr) and for both young and aged groups. This method ensured that each mouse contributed one mean firing rate value per time point, regardless of the number of neurons recorded. As a result, although the final dataset consisted of four averaged data points for both the young group and aged group per time point, the total number of neurons recorded exceeded the number of mice, due to instances where multiple neurons were successfully recorded within the same session. All the above parameters were computed for 5 s epochs (unless mentioned otherwise) using peak analysis tools incorporated in LabChart v8. The time spent in each state of NREM, REM, tREM and wake was determined by monitoring EEG and EMG within a 30 s window frame and scored manually per hour segment over 5 hrs of recording obtained from unanesthetized head-fixed mice in LabChart. The unit and EEG signals recorded in anesthetized and unanesthetized head-fixed mice were transferred to Igor Pro 8 (Wavemetrics) to make the original figures.

To quantify the phase relationships between the unit of cortical Up-states in association with EEG slow waves, we performed phase-locking analysis.^42^^,^^43^ The SO of EEG activity was filtered at 0.1-1 Hz. The filtered signals underwent Hilbert transformation to calculate the instantaneous phase values of the analytical signal. Spike phases were identified by extracting the instantaneous phase values at the corresponding unit spike times. To measure phase-locking, we calculated the mean phase and mean resultant length, which represent the angle and magnitude of the first trigonometric moment of the spike phases, respectively. Individual neurons' phase value distributions were visualized using phase histograms, while distributions of neurons were depicted using polar plots. In these polar plots, each neuron was represented by a vector indicating its mean phase, with the vector length displaying its mean resultant length (MRL), reflecting the strength of locking for young and aged mice. The analysis was performed in MATLAB (Figure S5).

#### Neurobiotin (Nb) staining

For searching Nb-labelled cells, frozen brain sections were cut at 25 mm on a freezing microtome. Serial sections through the SuM were stained for Nb using Cy3-conjugated streptavidin (SA-Cy3, 1:1000, Jackson ImmunoResearch Laboratories) and mounted on ProLong^TM^ glass antifade slides (Invitrogen) for examination by fluorescence microscopy. The obtained fluorescent NB^+^ cell image was transposed to the same location on the brain atlas to make Extended Data Figure 2B.

#### NMN administration and immunohistochemistry

To test the effect of NMN on EEG and the unit activity, PBS or saline and then NMN, diluted in either solution, were administered intraperitoneally (i.p) to C57BL/6J or *Slc12a8*-KO mice. As described previously,^44^ 300 mg/kg of NMN was administered to mice during recordings under urethane anesthesia or injected prior to the recordings in unanesthetized mice. For the chronic PBS and NMN administration under urethane anesthesia, PBS was administered via i.p. injection, followed by NMN ∼ 15 min later using the same route. For the long term administration, one group of mice received plain drinking water, while the other group received NMN supplemented in their drinking water, and the water bottles were changed weekly during the four weeks of NMN administration. In the fifth week, one mouse in each group was anesthetized with urethane per day, and EEG recordings were conducted for 70 minutes. Only the first hour of each recording was analyzed and used as representative data for Figures 1, 5A, 5B, and S1. All experiments were performed between 9:00 AM (ZT3) and 13:00 PM (ZT7). For head-fixed mice, saline or NMN was administered (i.p.) five minutes prior to the start of EEG recording. The recordings were conducted from 12:00 PM (ZT6) to 5:00 PM (ZT11).

For c-Fos immunohistochemistry, young (3-4 month old) C57BL/6 mice were injected (i.p.) with 300mg/kg NMN or PBS. 90 min later, mice were euthanized by CO_2_ and immediately transcardially perfused with PBS followed by 2% paraformaldehyde (PFA). The brains were removed and post-fixed with 2% PFA overnight at 4 °C, immersed in 20% sucrose in PBS at 4°C overnight until brains sank, and frozen by covering the brain with powdered dry ice. Fixed frozen brain tissue was sectioned using a cryostat at -20°C. Twenty-micrometer sections were mounted on Superfrost^TM^ Plus slides (12-550-15, Thermo Fisher). For immunohistochemistry, the sections were blocked with 5% goat serum (S-1000, Vector Laboratories) and 0.3% Triton-X100 (T8787, Sigma-Aldrich) in PBS for 1 hr, and incubated with a primary antibody diluted in blocking buffer (rabbit anti-c-fos, 1:1,000; 226003, Synaptic Systems) overnight at 4°C. The sections were then incubated with the secondary antibody, Alexa 647 anti-rabbit IgG (1:1,000; Jackson ImmunoResearch) for 1 hr at room temperature. Nuclei were stained with DAPI solution (D9542, Sigma-Aldrich). Images were acquired using fluorescent microscopy (DMi8, Leica Microsystems). The number of cFos-positive cells was quantified using ImageJ software (NIH, Bethesda, MD, USA).

#### Activity and novel object recognition tests

Behavioral tests were conducted in the Washington University Animal Behavior Core. All tests were conducted by a female experimenter blind to the genotypes of each mouse, and during the light phase of the light/dark cycle. For the 48-hr locomotor activity test, the mice were evaluated in transparent enclosures (47.6 x 25.4 x 20.6 cm high rat cages) with bedding, food and water, on a 12/12 light dark cycle. General activity variables of ambulation and rearing were collected over the 48-hr period using Motor Monitor software (Kinder Scientific, Inc). Mice were placed into the chambers at the beginning of the dark phase (6pm) on the first day. Recognition memory was evaluated using a novel object test. Each mouse was habituated to the test chamber over two days before the test sessions. For novel object recognition, during the sample trial, mice were placed into the familiar arena with two copies of the same object and allowed to explore for 10 min, and then returned to their home cage. A 10-min test trial was conducted following a 24-hr delay, when the mice were placed back in the test arena, where a novel object was presented along with the copy of the familiar object used during the sample trial. The amount of time each animal spent actively investigating the objects was scored using ANYMaze tracking software (Stoelting, Inc) with specified investigation zones 2 cm around the objects. Investigation was counted when the mouse’s nose is located within the investigation zones around the object.

#### RNAscope *in situ* hybridization

C57BL/6J mice were euthanized with CO_2_, and brains were perfused and processed as described above for c-Fos immunohistochemistry. RNAscope was performed using the RNAscope Multiplex Fluorescent Reagent Kit v2 (Advanced Cell Diagnostics, ACDBio) according to the manufacturer’s protocol. In brief, air-dried sections were washed with PBS and baked for 30 min at 60°C. Then, sections were fixed with 4% PFA/PBS for 15 min at 4°C. The slides were dehydrated with 50%, 70%, and 100% EtOH. After dehydration, the slides were treated with hydrogen peroxide solution for 10 min at room temperature. After washing with distilled water, the sections were dehydrated with 100% EtOH for 5 min and air-dried. The sections were treated with Protease III for 30 min at 40°C using a HybEZ oven, and rinsed with distilled water. The slides were then hybridized with RNAscope probes for 2 hr at 40°C using the oven. Probes for the following mRNAs were used (all from ACDBio): mm-Slc32a1 (Vgat; cat no. 319191), mm-Slc17a6 (Vglut2; cat no. 1170921), mm-Slc12a8 (cat no. 846731). The signal was amplified by subsequent incubation of Amp-1, Amp-2 and Amp-3 for 30, 30 and 15 min, respectively, at 40°C using the oven. Each incubation step was followed by two 2-min washes using RNAscope washing buffer. The signals were amplified with TSA Plus fluorophores (Akoya Biosciences), the slides were stained with DAPI solution, and coverslips were mounted using FluorSave (Millipore). Images were acquired using fluorescent microscopy (DMi8, Leica Microsystems) and quantification of *Slc12a8*-positive populations in the SuM was performed using CellProfiler.

#### Anterograde tracing and optogenetics

The Cre-dependent viral vectors AAV5-Ef1a-DIO-EYFP (#27056-AAV5) for anterograde tracing experiment and AAV1-Ef1a-DIO-hChR2(H134R)-eYFP-WPRE (#20298-AAV1) for optogenetic experiments were obtained from Addgene. Vector stocks were titered by rtPCR (Eppendorf Realplex) and virus at 1x10^12^ was used for injection.

For the tracing experiment, Vgat-Cre mice received unilateral injections of 30-50 nl of AAV5-Ef1a-DIO-EYFP into the SuM using a glass pipette. The coordinates targeting the SuM were AP= -2.3 mm, ML= ± 0.3 mm, DV= 4.8 mm. Brains were collected 3 weeks after virus injection and processed for IHC as described above using 4% PFA/PBS for perfusion and overnight fixation and 30% sucrose saturation before freezing. IHC was performed for GFP (ab13970, abcam, 1:500) on 20 μm sections. For IHC, brain sections were washed with Tris-buffered saline (TBS), then blocked with blocking buffer for 1 hr at room temperature (RT). Sections were then incubated with the primary GFP antibody in blocking buffer overnight at 4°C. Brain sections were then incubated with the secondary antibody in TBS for 1 hr. A DAPI solution (D9542, Sigma-Aldrich) was used to stain nuclei. Images were acquired using a fluorescent microscope (DMi8, Leica Microsystem) and processed using ImageJ (NIH, Bethesda, MD, USA).

For the optogenetics experiment to selectively activate SuM GABAergic neurons, Vgat-Cre mice were given bilateral injections of AAV1-Ef1a-DIO-hChR2(H134R)-eYFP-WPRE into the SuM using the same coordinates as for tracing. Mice were injected with 200nl AAV each side using a 0.5 μl Neuros syringe (65457-01, Hamilton Company). Optogenetics was performed 3 weeks post-surgery.

Photostimulation with blue light (473 nm) was applied through the optic fiber (∼100-μm core diameter, 0.22 NA, Thorlabs) using a CNI laser model (CNI optoelectronics technology, MBL-III-473-100 mW) connected to a TTL-gated power supply (PSU-III-FDA) which was then connected to a pulse signal generator (Master 8, AMPI) as in previous work.^31^ The optic fiber was lowered toward the SuM with angle (∼40o) (ML: 4.3 mm, AP: -2.3 mm) at the same bregma level as for the glass micropipette recording the SuM cells. Cortical cells were recorded in the cingulate cortex, using a glass micropipette placed at the coordinates (AP: -0.1 to -0.3 mm, ML: ∼ 0.5 mm, DV: 0.5 to 0.6 mm). The laser power for stimulation was set to ∼30 mW at the tip, which would correspond to ∼11 mW mm^−2^ at 1 mm from the tip (https://web.stanford.edu/group/dlab/cgi-bin/graph/chart.php) to reach the SuM neurons. The light intensity used lies within what is considered to be a safe range of ≤75 mW mm^−2^ for *in vivo* experiment.^33^ To find cells to record, light pulses of 50- or 100- ms duration were delivered every 2-3 seconds, and the unit was identified as a target cell using 5–15 ms to test whether the unit emitted a spike within a short light pulse as shown in previous. Units in association with EEG activity were also examined in the SuM or cortex for long (∼5 s) continuous light pulse and/or rhythmic light pulses at low theta frequency (∼200 ms pulse at 4 and 6 Hz) to the SuM of *Vgat*-Cre/ChR2 mice.

#### Chemogenetics (DREADD)

Stereotaxic bilateral injections of AAV5-hSyn-DIO-hM4D(Gi)-mCherry into the SuM of young (3 month old) Vgat-Cre male mice were performed, followed by a 3-week recovery period. EEG implantation for sleep recording was then conducted. Recordings were conducted in freely moving conditions for 24 hrs as described above, beginning at 6:00 AM (ZT0). On Day 1, mice received PBS (i.p.) at 9:00 AM (ZT3). On Day 2, DREADD Agonist 21 was administered to the same individuals (i.p., 1 mg/kg) at 9:00 AM (ZT3) (Figure 8A, right).

### Quantification and statistical analysis

Statistical analysis for anesthetized, unanesthetized head-fixed mice, and behavioral testing was carried out using GraphPad Prism 9. Measurement of differences between two or several groups were assessed using Student’s unpaired two-tailed t test or in some cases two-way repeated measures ANOVA, and comparisons among groups were performed using one-way repeated measures ANOVA followed by Tukey’s or Šidák post hoc tests. P values < 0.05 were considered statistically significant (∗p<0.05, ∗∗p<0.01, ∗∗∗p<0.001, ∗∗∗∗p<0.0001). Figures were prepared using Igor Pro 8, GraphPad Prism 9, MATLAB and Adobe Illustrator CC. Results are presented as mean ± SEM. All n values for unit recording data represent individual neurons. Statistical analysis for recording in freely moving mice was performed using SigmaStat 3.5 software.
