# Supplementary material for: Nicotinamide mononucleotide stimulates the activity of bursting slow-oscillation neurons in the supramammillary nucleus and enhances REM sleep
Source: iScience. 2026 Apr 15;29(5):115741. doi: 10.1016/j.isci.2026.115741 (PMC13187527; doi:10.1016/j.isci.2026.115741)
Supplement: Document S1. Figures S1–S8 [file mmc1.pdf]

iScience, Volume *29*

Supplemental information

Nicotinamide mononucleotide stimulates the activity of bursting slow-oscillation neurons in the supramammillary nucleus and enhances REM sleep

Youssouf Cissé, Cynthia S. Brace, Virginia Hsu, Carla M. Yuede, Nicholas Rensing, Michael Wong, and Shin-ichiro Imai

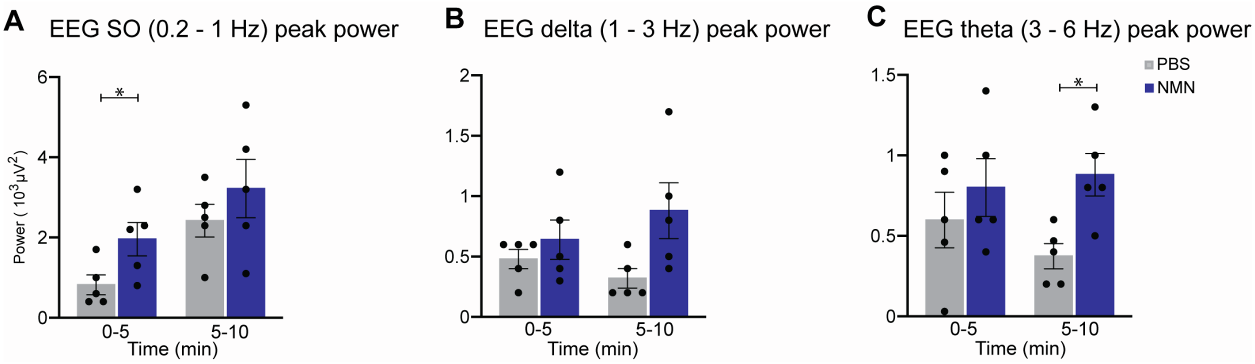

Figure S1 Related to Figure 1. NMN increases slow oscillation (SO) and theta power. (**A**) SO peak power shows an increase during 0-5 min time point and a trend of increase during 5-10 min time point. (**B**) Delta peak power shows a trend of an increase during 5-10 min time point. (**C**) Theta peak power shows a trend of an increase during 0-5 min and an increase during 5-10 min time point (two-way repeated measures ANOVA with Tukey's *post hoc* test, *p <0.05, n=5). Results are presented as mean ± SEM.

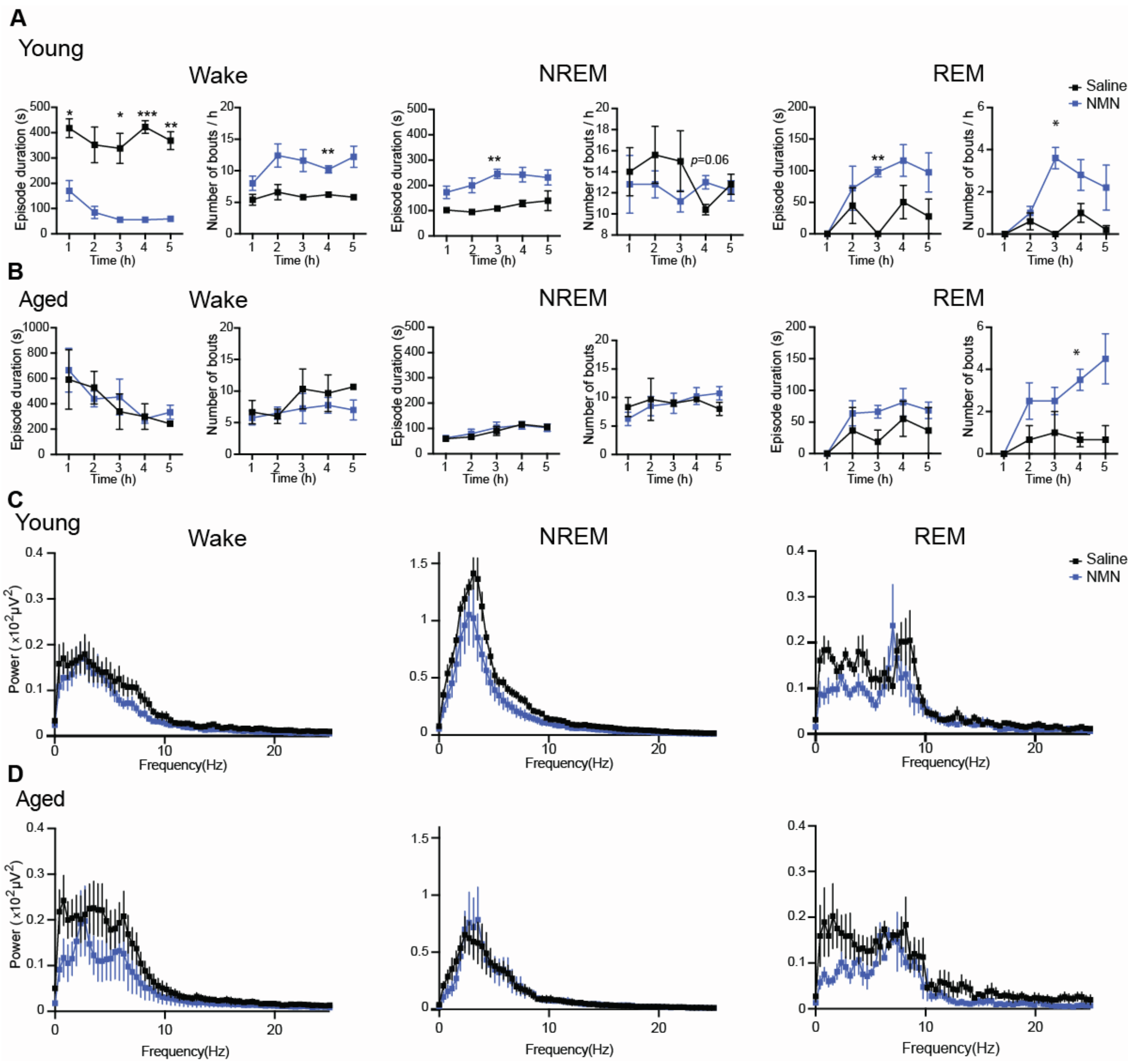

Figure S2. Related to Figure 2. Episode duration, number of bouts, and spectral power in saline- or NMN-treated mice during wake, NREM and REM sleep in young and aged mice. (A and B) Episode duration and number of bouts during wake, NREM and REM sleep for young (**A**) and aged (**B**) mice (*p<0.05, **p<0.01, ***p<0.001; two-way repeated measures ANOVA with Tukey's *post hoc* test, n=5-6 young mice; n=3-4 aged mice per group). (**C** and **D**) EEG power spectral during wake, NREM and REM for young (**C**) and aged (**D**) mice. Results are presented as mean ± SEM.

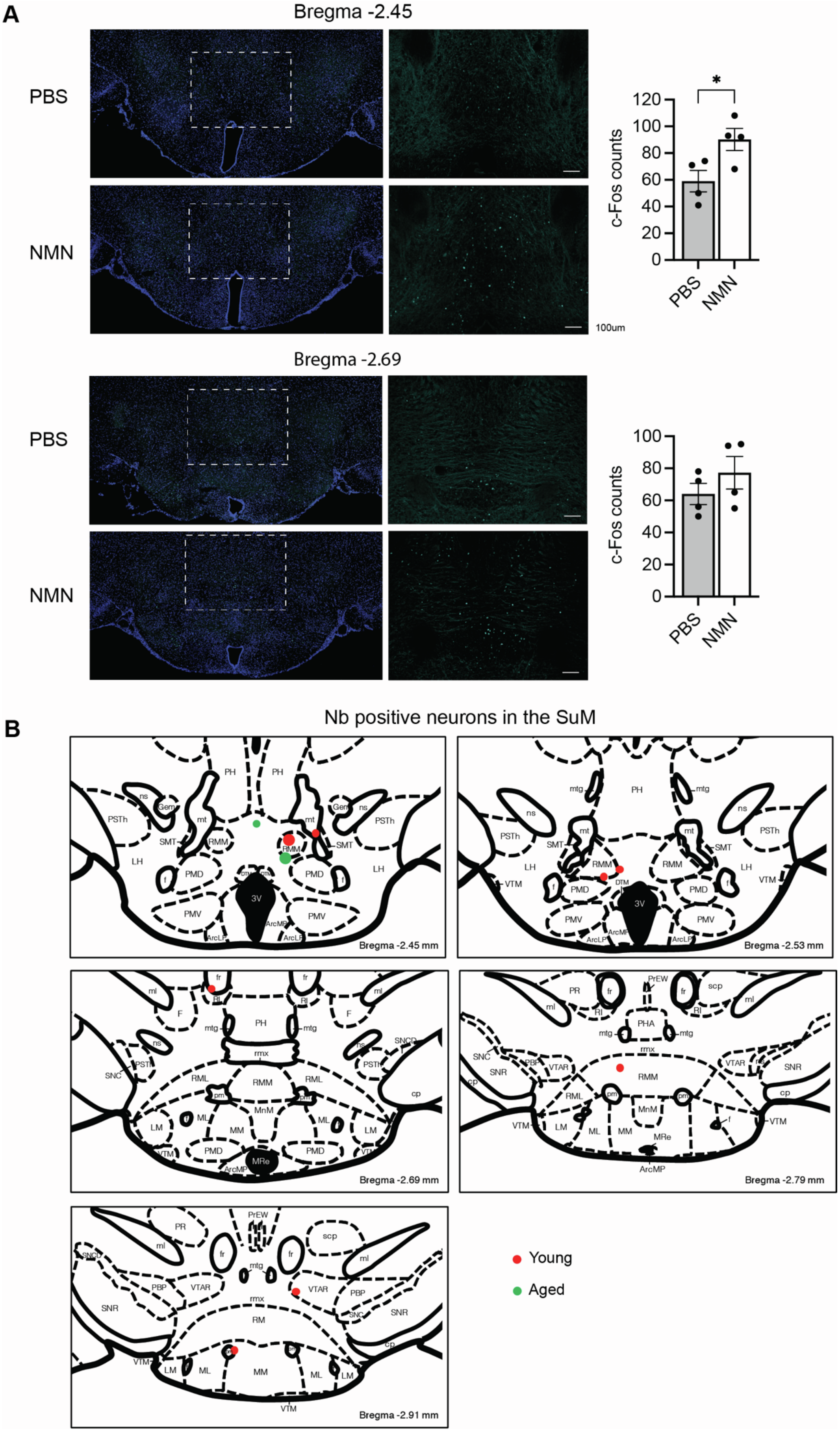
A
Bregma -2.45
PBS
NMN
100um
*
c-Fos counts
120
100
80
60
40
20
0
PBS
NMN
Bregma -2.69
PBS
NMN
c-Fos counts
100
80
60
40
20
0
PBS
NMN
B
Nb positive neurons in the SuM
Bregma -2.45 mm
Bregma -2.53 mm
Bregma -2.69 mm
Bregma -2.79 mm
Bregma -2.91 mm
Young
Aged

Figure S3. Related to Figure 3. NMN administration induces c-Fos expression in the SuM, and recorded neurobiotin (Nb)-labeled cells are located in the anterior part of the SuM in young mice. (**A**) Immunochemistry of c-Fos in the SuM in PBS- and NMN-administered mice at bregma -2.45mm (top panels) and bregma -2.69mm (bottom panels), and c-Fos quantification (right) (*$p < 0.05$, Student's t test, n=4 mice per group). Results are presented as mean ± SEM. (**B**) Location of Nb-labeled neurons throughout the SuM. The larger circles (red and green) at bregma -2.45mm are the representative neurons shown in Figure 3.

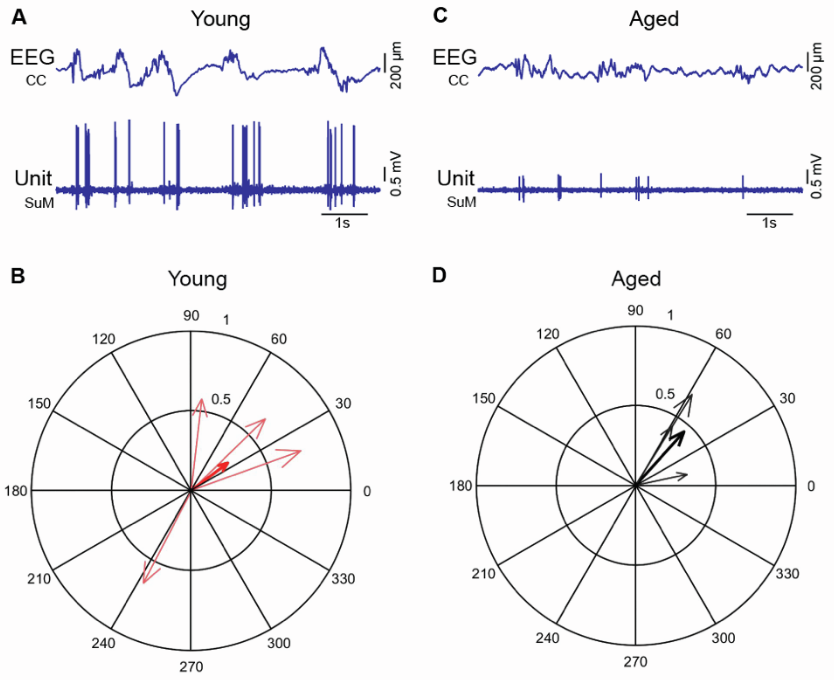

Cell ID	Degrees	MRL	P
Y-200326	-117.4	0.66	4.21E-37
Y-210127	83.6	0.58	6.16E-39
Y-210128	20	0.73	1.60E-37
Y-210203	44.1	0.64	3.10E-36

Cell ID	Degrees	MRL	P
A-210114	58.4	0.67	6.42E-08
A-210115	11.4	0.33	6.16E-04
A-210121	58.4	0.43	1.45E-10

Figure S4. Related to Figure 3. Phase locking of single units to the slow oscillation in young and aged mice. Units with EEG during SO were analyzed for young (**A, B**) and aged (**C, D**) mice. Polar plots show preferred phases of SO for individual units (thin arrows), and the group mean resultant vectors (thick arrows) are shown for young (**B**) and aged (**D**) mice. Arrow angle indicates the unit's preferred phase (0 deg, peak; 180 deg, trough); arrow length is the resultant length (vector strength, 0–1; concentric rings mark 0.5 and 1). All units plotted were significantly phase-modulated by the Rayleigh test for non-uniformity (*$p < 0.05$, 60s epochs per unit, n=4 from young, and 30s epochs, n=3 from aged mice). The group mean direction (angle of the thick arrow) did not differ between young and aged mice, as assessed with the Watson–Williams circular ANOVA ($p = 0.97$).

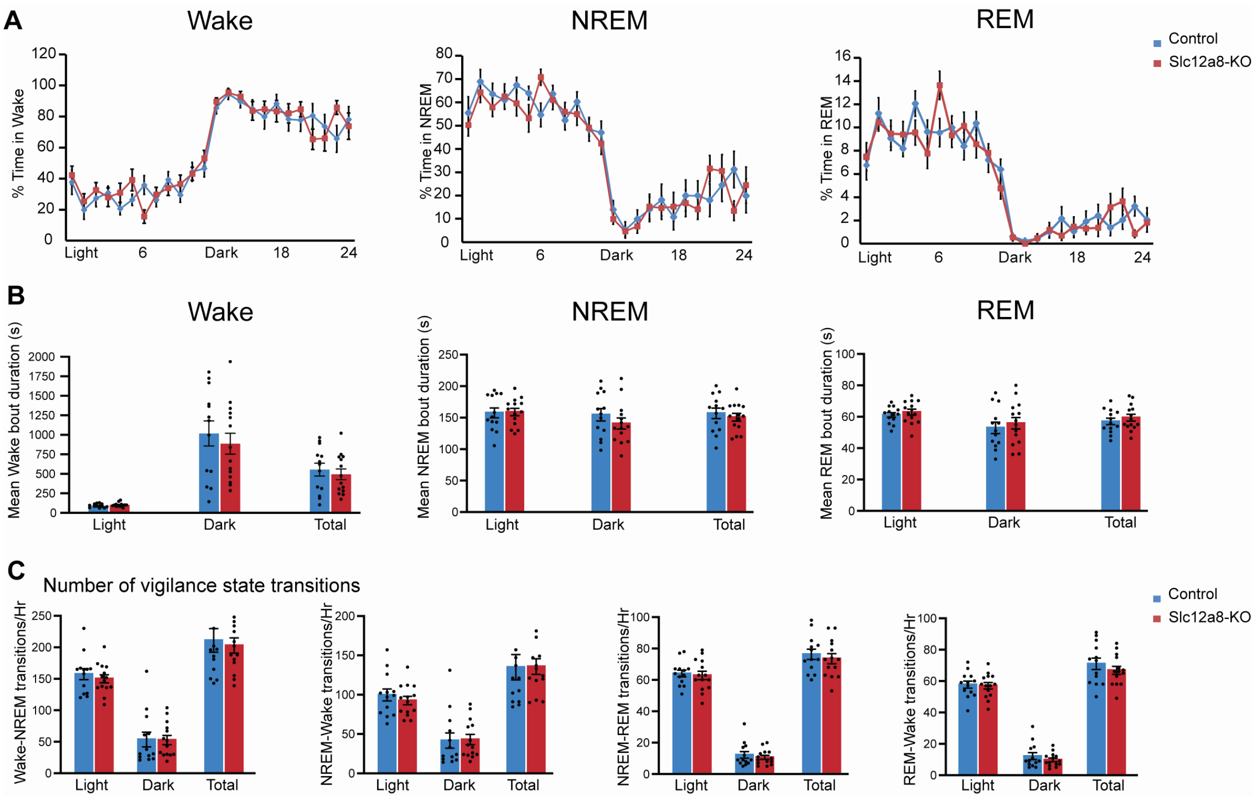

Figure S5. Related to Figure 5. Percentages of time spent, mean bout duration, and number of vigilance state transitions for Wake, NREM and REM sleep in control and *Slc12a8*-KO mice. (**A**) The percentages of time spent in Wake, NREM and REM sleep. (**B and C**) Mean bout durations **(B)** and numbers of vigilance state transitions during Wake, NREM and REM (**C**). (n=13-14 mice per each group). Results are presented as mean ± SEM.

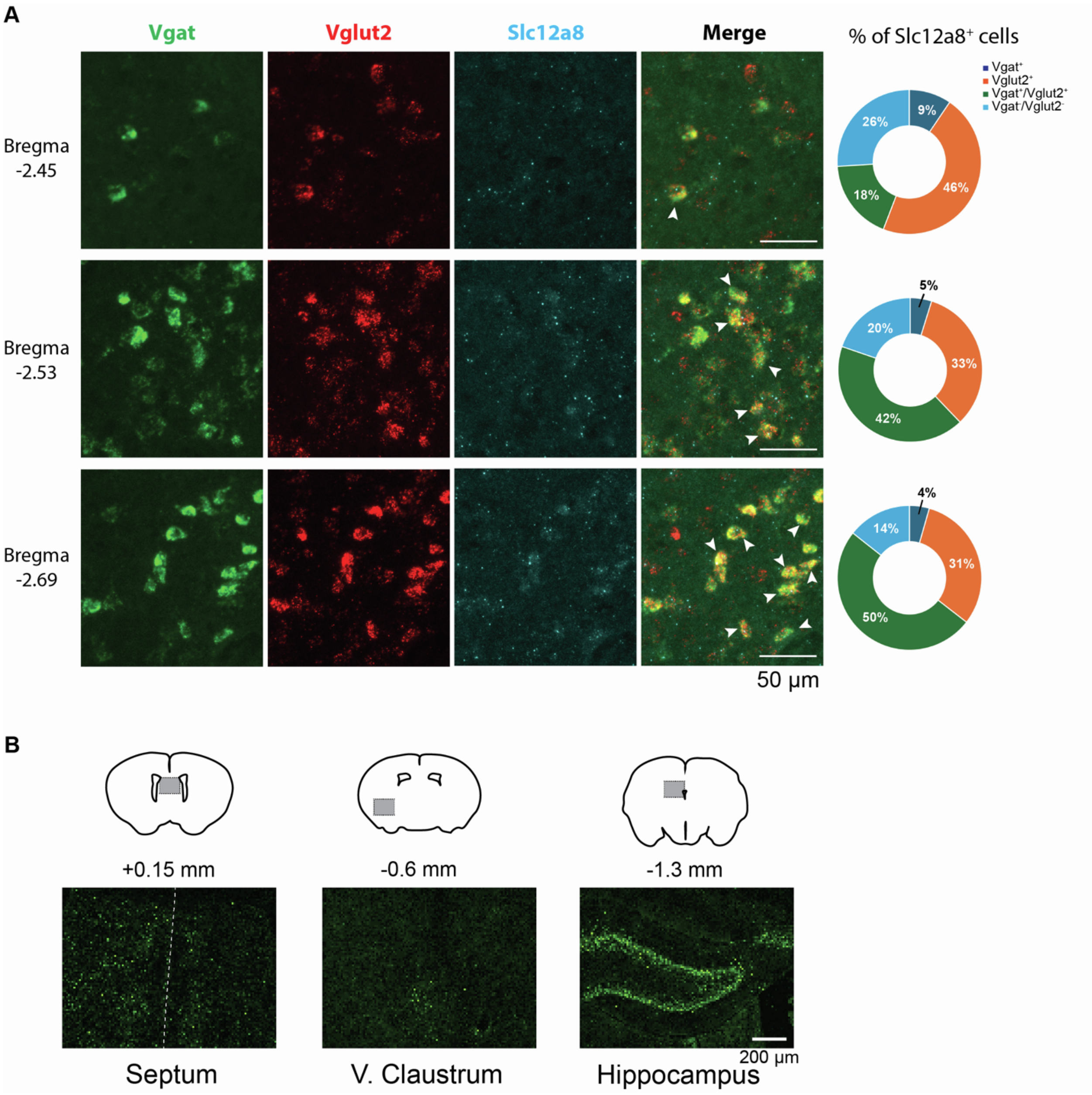

Figure S6. Related to Figure 6. *Slc12a8* expression in glutamatergic/GABAergic double-positive neurons in the SuM and other projection sites for the SuM GABAergic neurons. (**A**) RNAscope images for *Vgat* (green), *Vglut2* (red) and *Slc12a8* (blue) in the SuM of C57BL/6J mice are shown, and pie charts show the percentages of *Slc12a8*-positive cells that co-express *Vgat*, *Vglut2*, both *Vgat* and *Vglut2*, or neither at each bregma. White arrowheads indicate glutamatergic/GABAergic double-positive neurons that express *Slc12a8*. Scale bars are 50 µm. (**B**) Anterograde tracing from SuM GABAergic neurons identified three additional projection sites: the septum, ventral claustrum, and hippocampus. Scale bars are 200 µm.

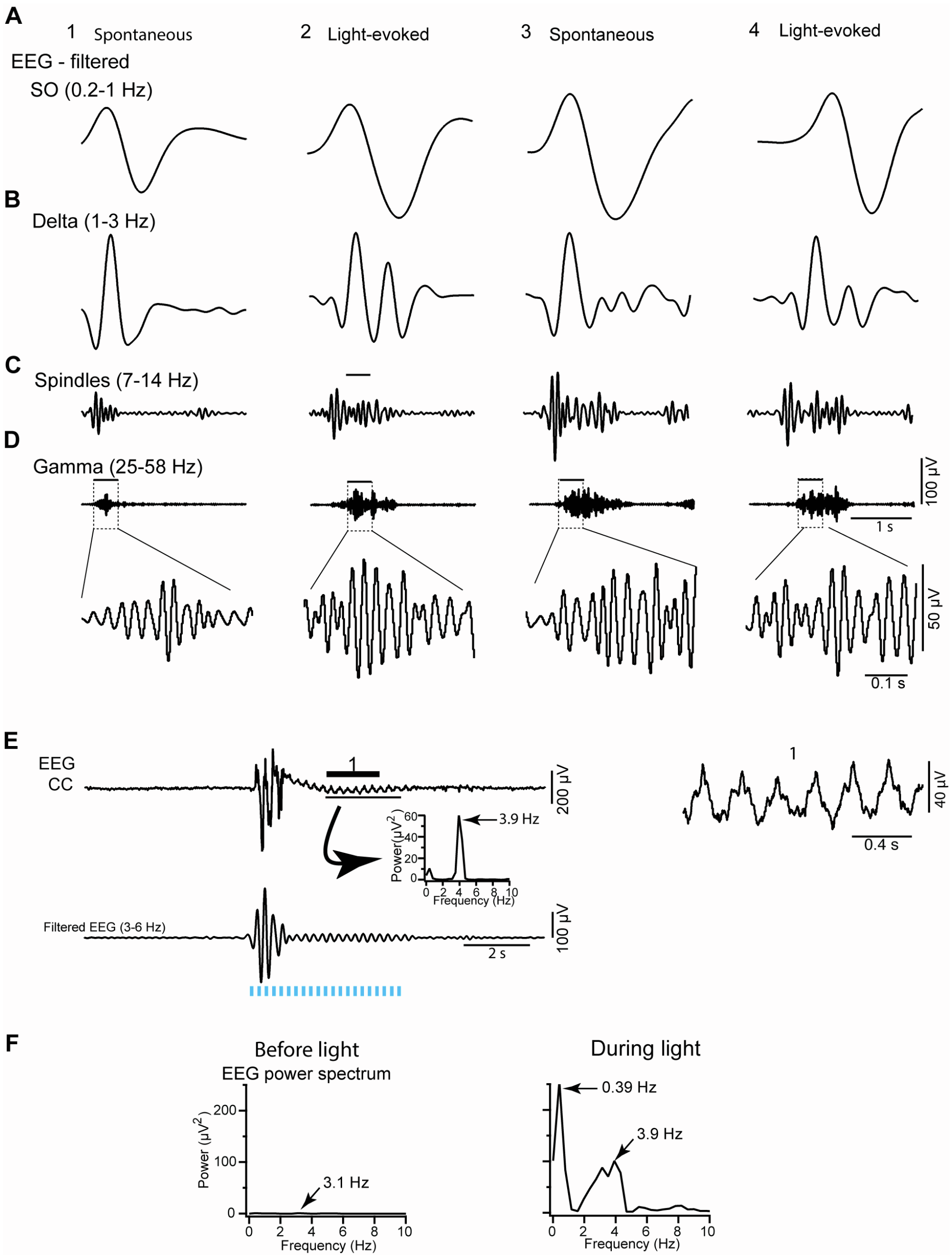

Figure S7. Related to Figure 6. Rhythmic light pulse stimulation applied to the SuM GABAergic neurons induces slow oscillation with their corresponding filtered traces, and 4 Hz pulse generates rhythmic theta activities. (**A-D**) SO (**A**), delta (**B**), spindles (**C**) and gamma (**D**) frequency bands were found to be nested on the EEG recording shown in Figure 6E. Periods from filtered gamma traces are expanded at the bottom **(D)**. (**E**) Cortical EEG with its filtered trace is displayed. The rhythmic light induces a large amplitude slow wave followed by rhythmic theta activity during the stimulation, as shown in the expanded segment 1 and its corresponding power spectral indicated by arrow with a peak at 3.9 Hz. (**F**) EEG power spectral showing peaks at 0.39 Hz and 3.9 Hz of SO and theta, respectively, during light stimulation is shown.

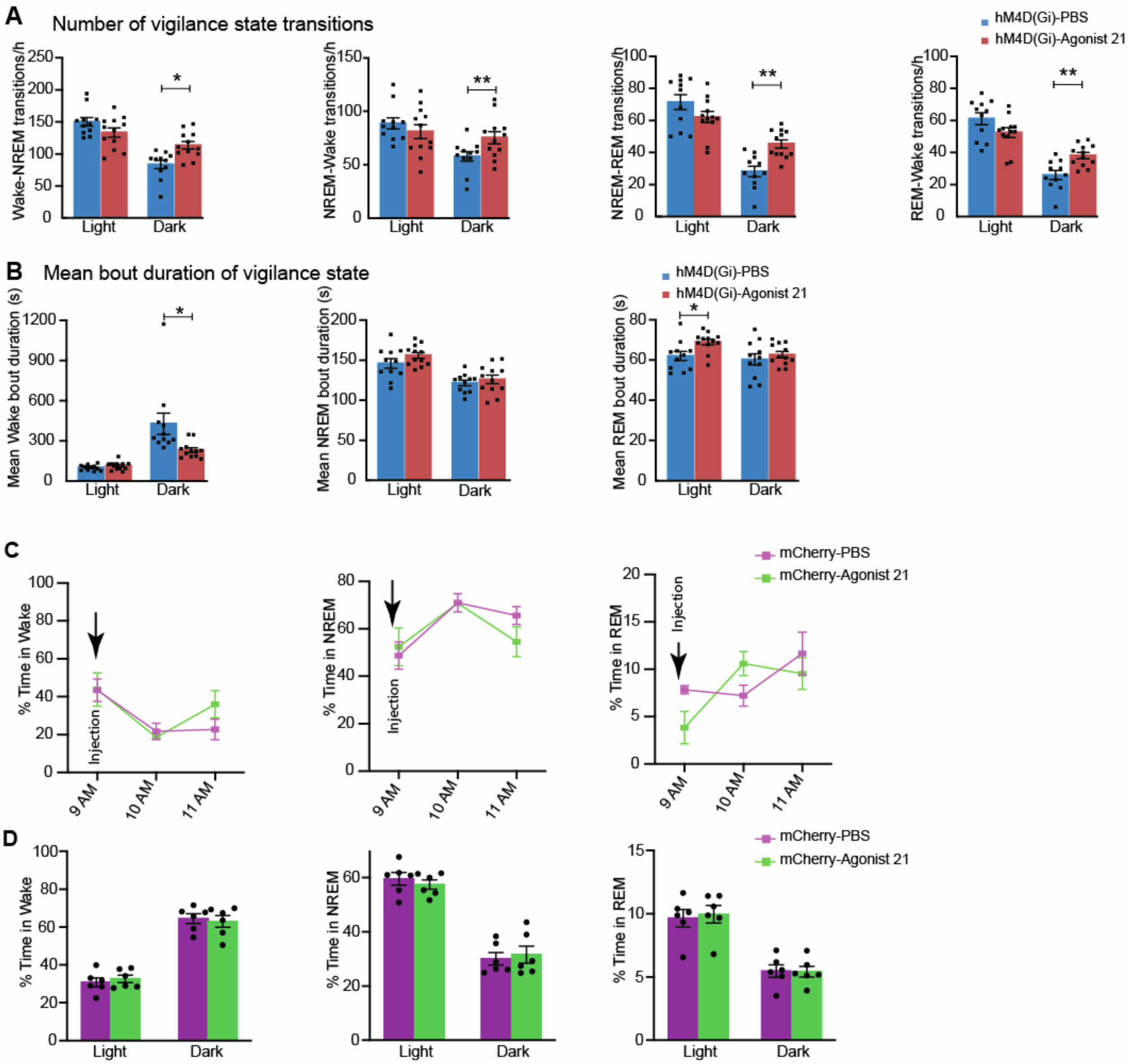

Figure S8. Related to Figure 8. DREADD inhibition of the SuM GABAergic neurons increases the number of vigilance state switching during the dark time, and Agonist 21 treatment has no effect in mCherry *Vgat*-Cre mice. (**A**, **B**) Number of vigilance state transitions and mean bout duration during light and dark times throughout 24 h recording (n=12 mice per group; *$p<0.05$, **$p<0.01$, paired, two-tailed Student's t test). (**C, D**) The percentages of time spent in wake, NREM and REM sleep during 2h post-treatment (**C**) and during light and dark times (**D**) in mCherry *Vgat*-Cre mice (n=6 mice per group). Results are presented as mean ± SEM.
